# The role of host genetic factors in respiratory tract infectious diseases: systematic review, meta-analyses and field synopsis

**DOI:** 10.1038/srep16119

**Published:** 2015-11-03

**Authors:** Inga Patarčić, Andrea Gelemanović, Mirna Kirin, Ivana Kolčić, Evropi Theodoratou, Kenneth J. Baillie, Menno D. de Jong, Igor Rudan, Harry Campbell, Ozren Polašek

**Affiliations:** 1 Department of Public Health, University of Split School of Medicine, Split, Croatia; 2 Centre for Global Health Research, Usher Institute of Population Health Sciences and Informatics , University of Edinburgh, Edinburgh, UK; 3 Roslin Institute, University of Edinburgh, Midlothian, UK; 4 Intensive Care Unit, Royal Infirmary of Edinburgh, Edinburgh, UK; 5 Department of Medical Microbiology, Academic Medical Centre, University of Amsterdam, Amsterdam, The Netherlands

## Abstract

Host genetic factors have frequently been implicated in respiratory infectious diseases, often with inconsistent results in replication studies. We identified 386 studies from the total of 24,823 studies identified in a systematic search of four bibliographic databases. We performed meta-analyses of studies on tuberculosis, influenza, respiratory syncytial virus, SARS-Coronavirus and pneumonia. One single-nucleotide polymorphism from *IL4* gene was significant for pooled respiratory infections (rs2070874; 1.66 [1.29–2.14]). We also detected an association of *TLR2* gene with tuberculosis (rs5743708; 3.19 [2.03–5.02]). Subset analyses identified *CCL2* as an additional risk factor for tuberculosis (rs1024611; OR = 0.79 [0.72–0.88]). The *IL4*-*TLR2*-*CCL2* axis could be a highly interesting target for translation towards clinical use. However, this conclusion is based on low credibility of evidence - almost 95% of all identified studies had strong risk of bias or confounding. Future studies must build upon larger-scale collaborations, but also strictly adhere to the highest evidence-based principles in study design, in order to reduce research waste and provide clinically translatable evidence.

Infectious diseases are characterized by a number of unique features - they rely on a single agent as a cause, they can be transmitted from one person to another and cause epidemics, and they have had a strong impact on human evolution^1^,^2^. In addition, infectious diseases can be eradicated, but also new ones may emerge, creating a dynamic stage for human-infection interplay^3^.

Even the most basic insight into the infectious disease occurrence and outcomes suggests strong inter-individual differences, commonly attributable to the host genetics profile. Adoption, twin and heritability studies provided the first line of evidence that corroborate this^4^,^5^, leading to an increased interest in understanding of genetic background of infectious disease in the last decade of the 20^th^ century^6^,^7^. As a result, over 4,000 candidate-gene studies were published between 2001 and 2010, often focusing on respiratory infections and especially on tuberculosis^8^. However, these studies often provided conflicting results^9^,^10^. Some of the problems of such studies included low study power, high risk of publication bias, differences in study designs, especially cases and controls recruitment schemes, which altogether led to often bias and confounding, genotyping inaccuracies, and substantial problems in phenotype definition, especially in the case of tuberculosis^11^,^12^,^13^. The entire field was plagued by very unfavourable effect-to-bias ratios, a situation in which the magnitude of bias exceeds that of the sought effect^14^. Not even the use of hypothesis-free approaches, like genome-wide association studies^15^, managed to spark a progress of the field, indicated as a lack of replication of results from such studies^10^,^16^,^17^,^18^. Unfortunately, insights derived from the fields of clinical infectology, microbiology, immunology, epidemiology, as well as clinical, evolutionary and population genetics remained largely isolated from one another^19^,^20^,^21^, preventing systematic understanding of the entire field.

The interest in host factors associated with respiratory infectious diseases stems from their ability to cause epidemics and pandemics. The infamous Spanish influenza of 1918 has been implicated as the largest ever recorded pandemic, causing an estimated 25–100 millions of deaths^22^,^23^. On the other hand, tuberculosis is an example of highly adaptive pathogen, which managed to coevolve with humans, possibly as far as the initial waves of human migrations out of Africa^24^,^25^,^26^. The 2013 estimates suggested that 9 million of people suffered from it globally, with 1.5 million of attributable deaths annually and possibly as much as a third of all living humans being latent carriers^27^. Pneumonia remains to be one of the main childhood mortality causes, with an estimated 120 million episodes and 1.3 million of lethal outcomes globally^28^. Similarly, respiratory syncytial virus (RSV) is attributable for 33.8 million episodes of newly diagnosed acute lower respiratory infections worldwide in children under 5 years, with at least 3.4 million episodes of severe cases requiring hospital admission^29^. Finally, SARS-Coronavirus outbreak in 2003 was an example of how quickly a novel respiratory pathogen can spread on a global scale^30^,^31^.

Understanding the host genetic side could be an invaluable tool in clinical medicine, especially because susceptibility to an infectious agent lies at least partly hidden or masked in inborn errors or immune response^32^,^33^. This renders infectious diseases a high-ranking research priority, having in mind the mobility of modern human population and the ever changing pathogen nature. Therefore, the aim of this study was to provide a field synopsis and systematic review with meta-analyses of host genetic susceptibility to respiratory infectious diseases. A special focus was set on the comparative analysis of different clinical presentations and pathogens, aiming to increase the understanding of both shared and specific disease pathways that could have been identified on the basis of previously published candidate gene studies. We also provide a critical overview of the entire field and generalized framework for improvement of future studies in infect*-omics* - application of genomics, proteomics metabolomics or other *omics* technologies in understanding of the infectious disease development, progression and outcome.

## Results

**Figure 1:**
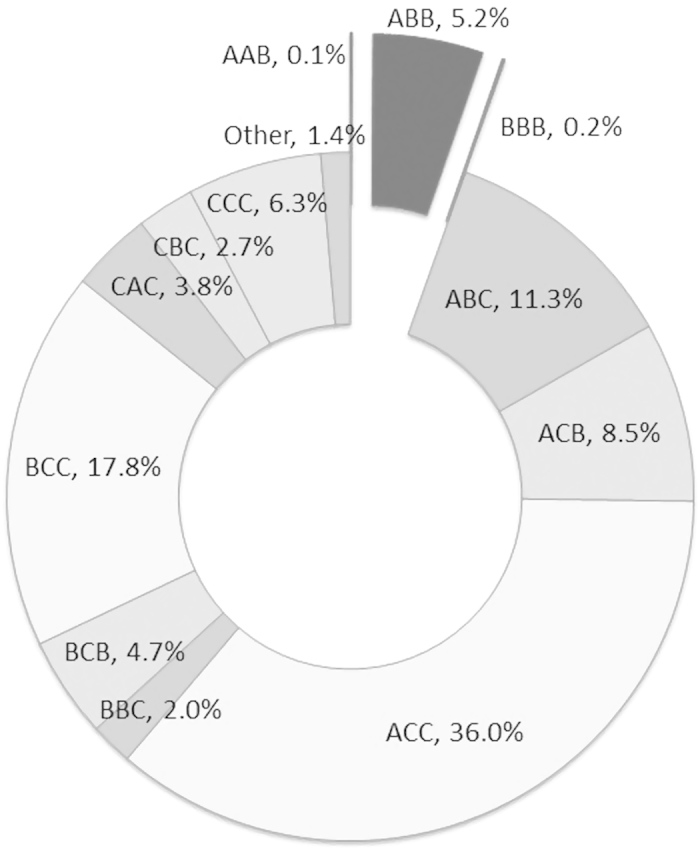
Quality score assessment, based on confounding, selection bias and information bias risk, based on 1,963 data points from RISE database (only data points with all three CSI domain scores were analysed here).

A total of 2,209 data points were extracted from 386 studies. The majority of data addressed tuberculosis (1,417 data points; 64.1%), followed by RSV (285; 12.9%), SARS-Coronavirus (198; 9.0%) and influenza (84; 3.8%), pneumonia (172; 7.8%), while the rest were on other respiratory infections such as otitis media and bronchitis (53; 2.4%). There were 274 different genes in the data, with most frequent results for *TNFA* (123; 5.6%), *IL10* (117; 5.3%) and *SLC11A1* (92; 4.2%). A total of 949 distinctive markers were recorded in the database, but only 89 met the inclusion criteria of at least four data points available per marker, and were used in the subsequent meta-analysis. Out of 1,963 data points with all three CSI score domains scored, only 107 data points (5.5%) had credible CSI score (having only A or B grades), while the remaining 94.5% of data points had at least one C grade and were thus considered to have weak credibility (Fig. 1). Disease-specific CSI profiles indicated better quality of studies on pneumonia, influenza and tuberculosis, while CSI scores were lower in case of SARS-Coronavirus and respiratory syncytial virus (RSV) (Supplementary Figure 1).

Among investigated data points, a total of 1,952 were attributable to infectious disease susceptibility (88.4%), 172 to disease severity (by comparing severe vs. mild disease cases; 7.8%) and 38 to mortality (1.7%), while the remaining studies were based on comparison of tuberculosis skin-test positivity results with controls. We performed a total of 515 disease susceptibility meta-analysis in all four genetic models, with a total of 86 nominally significant results (Supplementary Tables 3–6). However, only two genes retained noteworthiness for the mid/low BFDP level (Table 1). The first one was *IL4* gene, for pooled result in allelic model (rs2070874; OR = 0.79, 95% CI [0.70–0.89]; Supplementary Figure 2), and pooled result in recessive model (rs2070874; 1.66 [1.29–2.14]; Supplementary Figure 3). The second one was *TLR2* gene for tuberculosis in allelic model (rs5743708; 3.19 [2.03–5.02]; Supplementary Figure 4). We did not detect strong traces of publication bias in all meta-analyses (plots available in RISEdb website).

Subset analysis of methodologically better studies (with controls of known exposure to the pathogen) revealed five nominally significant results, with a single noteworthy result (Supplementary Table 7) - *CCL2* gene (rs1024611; OR = 0.79 [0.72–0.88]; Table 1). There were no significant results for severity models (Supplementary Table 7), while we did not perform mortality meta-analysis due to lack of at least four published studies for any marker.

We identified a total of 19 family and linkage-based studies, which were not included in the meta-analysis due to methodological restraints. These studies covered either broad genome regions on chromosomes 3–7, 10–12, 15, 17, 19, 20, X, or specific genes such as *SLC11A1* (*NRAMP1*), *IL8*, *CCR5*, *IL12RB1*, *SP*-*A, SP-D*, *SLC22A4*, *SCL22A5*, *RAD50*, *FBOX11* and *EVI1* gene (Supplementary Table 8). We also identified 11 GWAS studies; seven were on tuberculosis, three on otitis media and one on influenza. Two genome-wide significant results were recorded for tuberculosis, belonging to *WT1* gene and *ASAP1* gene (Supplementary Table 9). We also identified 54 published meta-analyses, of which the majority addressed genetic susceptibility to tuberculosis. However, these meta-analyses investigated infection susceptibility in much wider set of clinical appearances, often with manifestations not restricted to respiratory tract, and were thus not directly comparable to this study.

## Discussion

This is the first extensive, systematic review and meta-analysis of all published studies that were addressing host genetic factors implicated in five common respiratory tract infectious diseases - tuberculosis, influenza, respiratory syncytial virus (RSV), SARS-Coronavirus and pneumonia. Until now, majority of published studies and meta-analyses were based on one pathogen - one polymorphism approach, focusing on pathogen susceptibility. Only a handful of published studies compared different respiratory tract diseases. In addition, majority of previously published meta-analyses addressed tuberculosis, and none was found for SARS-Coronavirus, RSV and influenza. Therefore, by developing this entire resource we enabled not only disease-specific analyses, but also analyses of the shared (pooled) infectious disease mechanisms in the respiratory system - all in the stringent evidence-based manner.

In total, we found two results that withstood multiple testing correction: *IL4* in pooled model and *TLR2* for tuberculosis. Additionally, we established a role of *CCL2* in tuberculosis, by including only a subset of methodologically better studies, where controls were of known exposure.

*IL4* has previously been described to have a pivotal role in shaping the nature of immune response, by promoting and stimulating both T-cell and B-cell differentiation^34^. It provides a balance between Th1 and Th2 response, and therefore alteration of its function may substantially affect immune response^34^. Most commonly reported such alteration is associated with increased risk of atopy and allergies^35^. Furthermore, *IL4* seems to have a direct role in infectious disease outcome - *IL4*-deficient mice were more susceptible to *Legionella pneumophila* and had increased mortality rates compared to controls^36^. This was the single result that was significant in pooled analyses, and marginal in disease-specific meta-analyses. This reflects the need for studies with more statistical power, but also suggests that this is one of the most likely translatable results towards clinical application across the wider spectrum of respiratory infectious diseases.

*TLR2* is a member of large family of genes involved in pathogen recognition and signalling cascade of innate immune response^37^. Previous studies have suggested its role in the susceptibility to tuberculosis^38^,^39^,^40^, but also in other bacteria such as *Mycoplasma*, *Mycobacterium*, *Neisseria* and other Gram-positive bacteria^41^. Interestingly, despite being closely linked to *CD14* mechanisms^37^, the results of this study showed that *CD14* had no association with tuberculosis, while *TLR2* had a very strong result, with odds ratio of 3.2. This finding could point towards a specific role of *TLR2* in tuberculosis, with weaker association or even lack of such association in other elements of the immune response. Interestingly, *TLR2* effects seem to be very context-specific, as the absence of *TLR2* in knock-out mice did not affect their clinical outcomes, nor did it seem to have a role in secondary infections^42^. This suggests that the exact mechanisms of its function are still elusive, and that more focused studies are needed, taking into account the exact pathophysiological processes in patients with tuberculosis.

*CCL2* gene has been associated with diseases that include monocytic infiltrations^43^, migration and retention of monocytes in particular locations^44^, and consequently in granuloma formation^45^,^46^,^47^. As such, its role in tuberculosis has been described previously^48^,^49^, with notable ethnic-specific differences^50^. In this study, *CCL2* gene was significant only in a subset analysis, where studies with exclusively exposed controls were sub-selected. This clearly demonstrates that methodological improvements can have positive effect on gene discovery. Very complex genetic architecture of infectious diseases development, coupled with errors and omissions in primary study designs, might explain this accumulation of large amount of poorly usable evidence. Therefore, one of the main conclusions of this study is that the best way forward is through improvement of primary studies and reduction of research waste.

An overview of family and linkage-based studies suggested frequent differences in study designs, marked by dissimilarities in phenotype definitions, marker types and analytic approaches. Nevertheless, the association of tuberculosis with *SLC11A1* was replicated in two studies^51^,^52^, with a notable exception in the third one (Supplementary Table 8)^53^. There was no overlap of candidate-gene studies and GWAS^10^,^54^, thus prohibiting comparative analysis. All GWAS studies provided a two significant and replicated result, located in an intergenic region near *WT1* gene (rs2057178)^16^,^55^ and ASAP1 gene (rs10956514)^56^. Other reported signals were not replicated in independent cohorts (Supplementary Table 9). This is likely a consequence of largely underpowered study designs (with sample sizes rarely exceeding 1,000 cases or controls), but more importantly methodological problems that were carried over from the candidate-gene study designs. Up-scaling the study in terms of genetic resolution (from single marker or just a few markers to thousands and hundreds of thousands of markers in GWAS), increase in the sample size and use of more advanced analytic methods will likely not provide a substantial step forward before we manage to overcome methodological and study design limitations. In addition, we are faced with a high level of genetic complexity^57^, and duality between rare, Mendelian variants, which may confer complete immunity, but have very low prevalence in a population^58^, or common variants, which usually have much lower explained variance, and therefore have a limited capacity for clinical intervention. The principal step forward is to create larger-scale collaborations and enable data sharing, built upon the common framework for analysis, based on aligned protocols, larger-scale harmonizing and collaborative efforts^59^.

The results of this study fit into previously implied mechanisms of respiratory infections, where *CCL7*-*CCL2*-*CCR2* axis was described to have a critical role in *IL4* production and immune response and regulation in both fungal^60^ and viral infections^61^, as well as atherosclerosis^62^ and tumours^63^. Despite differences described from *in vitro* and *in vivo* studies^64^, this could be the most interesting translatable mechanism towards clinical medicine. In addition, *WT1* gene from GWAS studies^16^,^55^, implied in susceptibility to tuberculosis, has been described to be under control of *CCL2,* which seems to be having target-responsive cytocidal activity on *WT1*-specific mechanisms^65^. This mechanism could, at least partly, explain the relationship between some infections and tumours^66^. However, to clarify their relationship and provide a step forward, we must firstly be able to derive much better evidence from primary studies.

Primary study design was one of the main barriers for the further field development, with almost 95% of all extracted data points assessed as having strong risk of bias or confounding. The improvements must be embodied into evidence-based principles, and have to be undertaken in almost all domains of the field, including better clinical definitions and phenotyping, improved controls selection and diagnostics, appropriate statistical analysis, favourable study design, the use of novel molecular technologies and better reporting with larger sample sizes obtained through consortia development. Firstly, we need to improve phenotype definitions and criteria for diagnosis, especially in case of latent tuberculosis^13^,^67^. The use of proxy and extreme phenotypes, which was seen in HIV, with exposed uninfected subjects, long-term non-progressors, fast progressors and elite controllers^68^,^69^,^70^, might also provide an interesting step forward^71^. It was very common for authors to use the term “healthy” controls, but almost universally used dissimilar diagnostic process for classification of cases and controls. Thus, selection of controls and diagnostics process must be fundamentally improved and harmonized (Supplementary Table 10). A substantial effort must go towards inclusion of controls that were exposed to a pathogen, which is necessary for the disease development (but may not be sufficient). Data analysis must also be improved, by adhering to the best practices, including the use and report of odds ratios and confidence intervals as measures of association, and multiple testing correction^72^. Improvements can be made in the study design, where availability of finely phenotyped life-long cohorts and biobanks provides an opportunity to use this data in an understanding of life-long risk of developing respiratory infectious disease, such as tuberculosis. The use of novel molecular technologies, various *omics*, and their application to infectious disease development, progression and outcome (infect-*omics*) presents an unprecedented opportunity in this field^73^. However, these must be based on evidence-based, always include estimates of repeatability and focus on replication efforts in an unlinked population. Lastly, we must substantially improve results reporting. This refers to the structured manuscript preparation, reporting of all relevant information and manuscript preparation against a given set of criteria like STREGA^74^, STROBE-ME^75^ or STREIS, where appropriate^76^. This also extends to the need to develop individual-data public repositories and creation of large-scale consortia that share common standard-operating procedures, similarly to these in chronic disease genetic epidemiology. We do not neglect the proposed finer scale analyses of various population subsets^67^, but we consider improvements in study design to have much stronger potential impact. All these suggestions are applicable not only for genomics, but also for any type of infect–*omics* studies, defined as application of various -*omics* technologies in understanding of the infectious disease development, progression and outcome. In addition, we need to start developing framework of systematic reviews, where we need to compare disease-specific genes and pathways to disease-specific genes, as the final outcome, clinical disease, might be attributable to different sets of genes that make a person more susceptible to e.g. pneumonia regardless on the pathogen that is causing it. Only by comparing such complementary results and insights will we be able to better understand and harness this information in clinical care.

These results must be viewed in light of numerous limitations. We reviewed a very diverse field with no unified reporting scheme, suggesting that the search might have missed some relevant articles and data sources. In order to accommodate for this problem we performed extensive search supplemented by hand-search procedure described in Materials and methods section. Existence of wider-scale unpublished data and occasionally our inability to obtain raw data present another possible source of bias. This study also suffers from the limited input, due to exclusion of HLA and KIR markers, haplotypes and complete omission of any information on pathogen, thus providing rather limited insight into the field and possibly missing out some of the key element for more in-depth understanding^77^. Most of these limitations have very negative impact on effect-to-bias ratio, where bias may be several orders of magnitude greater than the size effects. We attempted to contrast this by the use of the stringent analytic approach in an evidence-based manner, including multiple testing correction, careful application of inclusion criteria and quality assessment scoring, coupled with use of random-effect meta-analysis. The study also suffers from inability to adjust the analysis for basic covariates, such as age, sex and comorbidity information. We also used a novel score for quality assessment that was developed within this study and was not validated before. We thus provided basic agreement indices, but wider use of this score would require an in-depth reliability assessment and validation. If we turn back to biological processes, earlier primary studies ignored microbiomes, while some novel studies are suggesting that lungs are not sterile^78^,^79^ and that respiratory tract microbiomes could have strong modifying effect on health and disease mechanisms. Despite all these limitations, we attempted to provide a field synopsis, supplemented by a generalized framework that could be useful not only for respiratory infectious disease genetics, but can be extended to all infectious diseases. However, these results are just the first step it an attempt to provide a reflection of just one side of the infectious disease story – the host. Only by combining the host genomics (and other *–omics*) information with pathogen *–omics* will we be able to truly advance this field and enable the development of personalized infectious disease medicine.

## Materials and Methods

### Protocol and registration

The review strategy and details were registered prospectively in PROSPERO as the record 2014:CRD42014009072.

### Eligibility criteria

We undertook an extensive and systematic search of all published studies related to host genetics implicated in development or outcome of respiratory tract infectious diseases. Instead of focusing on a single-disease approach, we aimed at delivering systematic evidence on all respiratory infections, including cases where a syndrome phenotype was reported, such as pneumonia, without the specific pathogen information.

### Information sources

We used three bibliographic databases; PubMed (http://www.ncbi.nlm.nih.gov/pubmed), Web of Science (http://wok.mimas.ac.uk) and Scopus (http://www.scopus.com). We also performed an additional search of the HuGe Literature Finder (http://www.hugenavigator.net), to identify articles that might have been missed in PubMed.

### Search

Two of the joint first authors performed the search and study selection, which was supervised by the last author. The last author also resolved any discrepancies in scoring system or data extraction process. The search was performed on May 06, 2014, with an update performed on August 25, 2015. After duplicates removal, the search yielded a total of 24,823 articles. All identified articles were firstly checked for inclusion by reading the Title and Abstract. This led to identification and exclusion of a total of 23,677 studies. The remaining 881 articles were supplemented by hand search of their references, producing an additional set of 265 articles. In total, we used a set of 1,146 articles that were read in full and screened against inclusion criteria (Supplementary Figure 5).

### Study selection

The study selection process was based on iterative steps. Firstly, the studies needed to report the number of cases and controls for every analysed genotype. Therefore, we initially excluded all of the family-based and linkage studies, as well as genome-wide association (GWAS) studies from this meta-analysis, due to inability to obtain complete set of GWAS results in the appropriate format. However, we retained these studies and provided their qualitative summary, in order to develop an inclusive field synopsis. Secondly, studies had to be based on biallelic single-nucleotide polymorphisms (SNP) or biallelic insertion-deletion marker type. Studies based on other marker types (including microsatellites or short tandem repeats), or those that reported aggregated haplotypes were excluded. We also excluded all studies that reported exclusively gene expression profiles without relevant genotyping information. We then limited the review to studies that were published during or after the year 2000 and were written in English language. Despite similarity of disease patterns and mechanisms, we excluded all animal and all *in vitro* studies and focused on susceptibility in previously healthy subjects. We also excluded studies that reported: any outcome in previously affected patients (either as a part of immunodeficiency syndromes or with any form of disease such as asthma, HIV/AIDS or other comorbidities; where reported), fungal infections (due to also very prevalent comorbidities), nosocomial infections (such as ventilator-related or hospital-acquired pneumonia), exclusively pathogen genotypes or host-pathogen interactions. Studies that reported non-respiratory infection sites (such as extra-pulmonary tuberculosis, meningitis or invasive pneumococcal disease) were also excluded, as the focus of this study was on respiratory infectious diseases. Lastly, we excluded all studies that were obviously reporting previously published results or were re-using the cases that has already been used elsewhere (when we managed to identify this), in order to remove redundant data or publications from meta-analysis. To ensure a systematic source of information, we additionally checked studies which provided insufficient information. A total of 142 e-mails were sent to authors of studies which we classified as having reported insufficient information, and asked for clarifications or raw data sharing. We also contacted all authors of identified GWAS studies to share their results. This entire process results in a total of 10 responses (4 rejections, 2 responses that did not meet the inclusion criteria and 4 cases of clarifications or shared raw data, which are listed in the Acknowledgements). In total, we used 386 articles in the data extraction process (Supplementary Figure 5).

### Data collection process and data items

A total of 48 pieces of information were extracted for every study (available in the on-line RISEdb – Respiratory Infection SuscEptibility database, available at http://www.prepare-europe.eu/risedb). All the data were extracted and entered into a database by two authors, and checked by the third, in order to ensure data integrity. Each identified study was indexed with a unique identification code (RISEdb ID). Some studies provided more than one data point (which corresponds to one marker - one study data entry), and were consequently represented with more than one data entry in our database. In all instances the data were extracted in the raw format as genotype counts, or were calculated from the reported percentages and total sample sizes where number of cases and controls was not available. When more cases or control groups were available in one study, we either pooled them or retained multiple data points from the same study, especially in case of having discovery and replication datasets. In addition, all data points were classified according to the underlying research question into three categories: studies that investigated infectious disease susceptibility, disease severity or disease mortality. In addition, we defined a subset of methodologically better studies, where controls were of known exposure to the pathogen.

### Risk of bias in individual studies

We developed a novel score for purposes of risk of bias assessment. As the methodological design defines, a study may be prone to three main domains of errors and bias – confounding, selection bias and information bias. These three domains were the basis for the score development, which was entitled Confounding-Selection-Information bias score - CSI (Supplementary Table 2). The elements of the score were developed on the basis of several previous studies^80^ and assessment scores, including Venice criteria for assessing cumulative epidemiologic evidence in genetic associations^81^, Newcastle-Ottawa case-control scale^82^, Cochrane risk of bias tool^83^, and previously established quality scores in genetic epidemiology^84^. Neither of these tools provided a good fit for the risk of bias assessment in observational studies in genetic epidemiology, and we therefore took elements of previous scales and derived a new one. In line with Venice criteria, we scored all three domains in three grades of credibility: high - graded as A; intermediate - B or weak – C. This scheme provided estimates ranging from the best AAA to worst CCC score, which was applied to every data point in RISE database (and not for the meta-analysis results, as the Venice criteria is intended to). Since information bias can affect several aspects of the study, we also divided information bias risk domain in three separate sub-scores, relating to cases and controls selection and genotyping procedures, with the worst of all three defining the overall information bias risk. All CSI scoring was done independently by two authors, and all discrepancies were settled by an agreement or input from a third author (there were 313 or 14.2% data points with initially dissimilar grades, providing good agreement with kappa coefficient of 0.717 and standard error for kappa of 0.011).

As an additional quality control check, we re-calculated Hardy-Weinberg test for every control set included in the analysis, using a two tailed chi-square test or an exact test, when appropriate. Those data points that failed HWE test at the level of P < 0.05 were downgraded to C in the CSI score (there were 225 out of 2,209, or 10.2% of all analysed data points that failed HWE), in order to reflect possible methodological limitations of failed HWE (which indicates both the risk for selection and/or information bias). Where cases from a single study were classified as moderate or severe, we did not determine Selection domain of CSI score (a total of 246 data points). In such instances we assigned 0 to selection bias risk, as there was no selection process involved, and second-stage analysis was performed

### Summary measures and synthesis of results

In the first step of meta-analysis we calculated all odds ratios anew from raw data, in order to provide a common analytic framework and reduce possible differences in data analysis. In order to better understand the genetic architecture underlying gene-disease association, we performed analyses using four separate genetic models: allelic, dominant, recessive and heterozygote advantage. Model selection was based on commonly used allelic model, two additional models that assume different inheritance effects (dominant and recessive), followed by the heterozygote advantage, as previous studies reported such results^85^,^86^,^87^. All calculations were therefore based on allele counts, rather than exact cases and controls counts. We compared the results of different models, in an attempt to provide broader insight into genetic architecture.

Minor allele frequencies in Homo sapiens Short Variation (GRCh38) data set were obtained from Ensembl Biomart on 08.12.2014^88^. We then performed a series of meta-analyses for all SNPs where four or more data points were available for a single SNP. We used random effects meta-analysis, assuming differences in designs and ethnic composition of individual studies. We also calculated fixed-effects model, in order to compare the results. We used I^2^ statistics and corresponding confidence intervals as a measure of heterogeneity.

In order to account for multiple testing as a result of numerous meta-analyses and different genetic models being performed, we used Bayesian false-discovery probability (BFDP)^72^. It was calculated for nominally significant results only, with a threshold of 0.2, using an online free version of the BFDP calculator (http://faculty.washington.edu/jonno/BFDP.xls). In addition, we calculated BFDP using two prior probabilities, with medium/low prior level (0.05 to E-03), consistent with a candidate gene; and very low prior level (E-04 to E-06), consistent with a random SNP. The advantage of this approach over the commonly used corrections is that it is not dependent on the number of tests performed, as it relies on odds ratios and confidence intervals for calculation. In addition, it provides conceptual improvement over the similar methods, such as false-positive report probability (FPRP)^89^. Due to substantial requirements for appropriate use of publication bias analysis methods^90^, we only examined publication bias visually.

All analyses were performed in R, version 2.15.3^91^. Meta-analysis was performed with package meta 3.2–0 and metabin, forest and funnel functions^92^. Hardy-Weinberg equilibrium testing was performed in HardyWeinberg_1.5.2 package^93^, with HWExactMat or HWChisqMat functions, based on the cell expected frequencies.

### Risk of bias across studies

For purpose of meta-analysis credibility assessment, we used Venice criteria^81^, in all instances of nominally significant results. Study power was assessed on the basis of sample sizes (with sample sizes of up to a 1,000 graded as C, 1,001–10,000 graded as B, and over 10,001 graded as A). Heterogeneity was based on I^2^ statistics (graded as A in case of 0–25%, B in case of 26–50% and C in case of I2 being over 50% ), while the third score domain was fixed as C (weak credibility), due to very prevalent risk of bias in primary studies.

## Additional Information

**How to cite this article**: Patarčić, I. *et al.* The role of host genetic factors in respiratory tract infectious diseases: systematic review, meta-analyses and field synopsis. *Sci. Rep.* **5**, 16119; doi: 10.1038/srep16119 (2015).

## Supplementary Material

Supplementary Information

## Figures and Tables

**Table 1:** Significant and noteworthy results of meta-analyses (the entire set of results is available in Supplementary Tables 3, 4 ,5 and 6).

Gene	Disease; genetic model	rs code	Heterozygote	Risk allele	N studies	N cases (alleles)	N Controls (alleles)	OR [95% CI]	P	I^2^ [95% CI]	Venice score	BFDP (med/low)	BFDP (very low)
IL4	Pooled result; allelic	rs2070874	CT	T	6	2908	6422	0.79 [0.70-0.89]	7.68E-05	0.06 [0.00–0.76]	BAC	0.063	0.779
IL4	Pooled result; recessive	rs2070874	CT	T	5	1059	2210	1.66 [1.29–2.14]	8.68E-05	0.00 [0.00–0.39]	BAC	0.128	0.886
TLR2	Tuberculosis; allelic	rs5743708	AG	A	6	3262	3124	3.19 [2.03–5.02]	4.91E-07	0.00 [0.00–0.63]	BAC	0.087	0.834
CCL2	Tuberculosis; heterozygote advantage, in exposed controls	rs1024611	AG	G	4	3066	3556	0.79 [0.72–0.88]	1.26E-05	0.00 [0.00–0.83]	BAC	0.014	0.426

Abbreviations used: TBC – tuberculosis, BFDP - Bayesian false-discovery probability.
