# Supplementary material for: The role of host genetic factors in respiratory tract infectious diseases: systematic review, meta-analyses and field synopsis
Source: Sci Rep. 2015 Nov 3;5:16119. doi: 10.1038/srep16119 (PMC4630784; doi:10.1038/srep16119)
Supplement: Supplementary Information [file srep16119-s1.doc]

**Supplementary information for manuscript:**

**The role of host genetic factors in respiratory tract infectious diseases: systematic review, meta-analyses and field synopsis**

Inga Patarčić, Andrea Gelemanović, Mirna Kirin, Ivana Kolčić, Evropi Theodoratou, Kenneth J. Baillie, Menno D. de Jong, Igor Rudan, Harry Campbell, Ozren Polašek

| 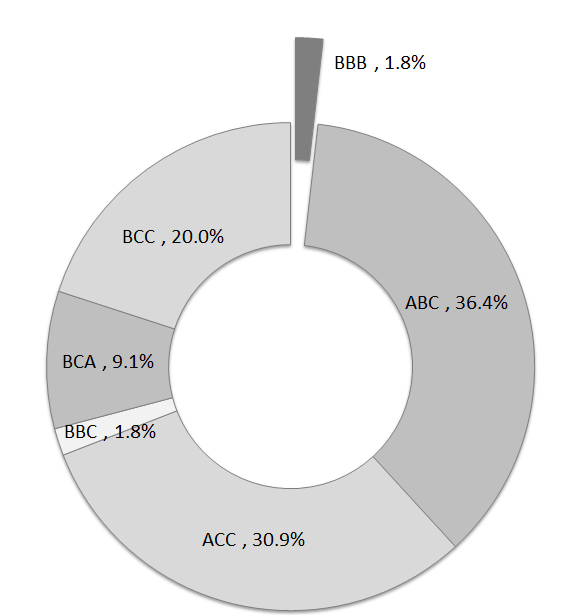  a) influenza | 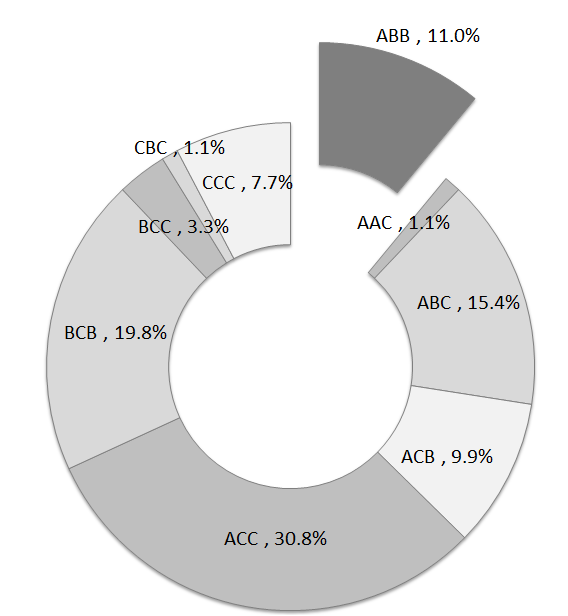  b) pneumonia |
| --- | --- |
| 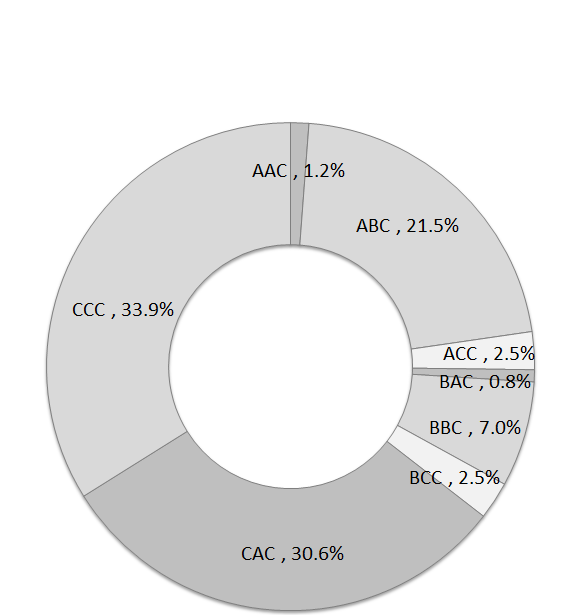  c) respiratory syncytial virus | 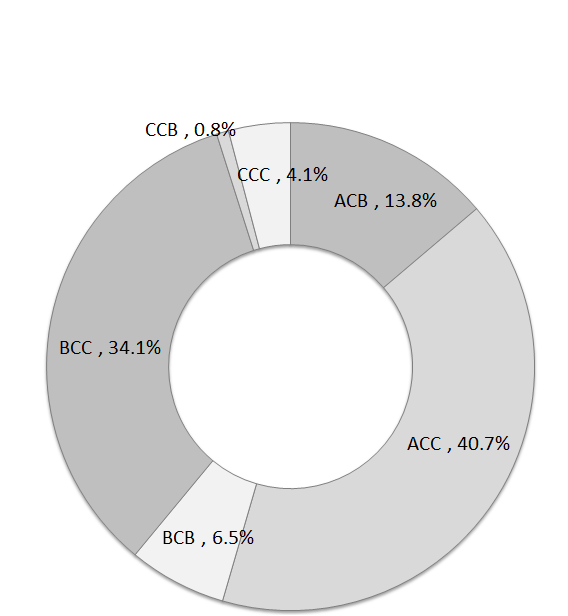  d) SARS-Coronavirus |
| 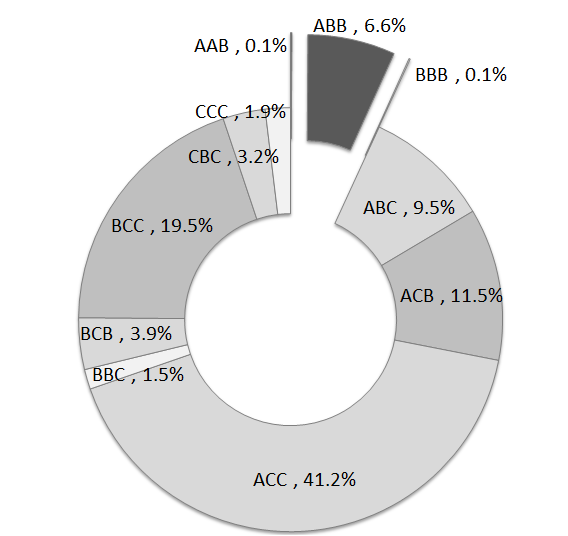  e) tuberculosis |  |

Supplementary Figure 1. Disease-specific CSI scores

Supplementary Table 1. Search term and inclusion criteria

| **PubMed (http://www.ncbi.nlm.nih.gov/pubmed/)** |
| --- |
| ("DNA"[MeSH Terms] OR SNP[All Fields] OR "polymorphism"[All Fields] OR "polymorphism, single nucleotide"[MeSH Terms] OR "single nucleotide polymorphism"[All Fields] OR "polymorphism, genetic"[MeSH Terms] OR "genetic polymorphism"[All Fields] OR "genes"[MeSH Terms] OR "genes"[All Fields] OR "gene"[All Fields] OR variant[All Fields] OR "genotype"[MeSH Terms] OR "alleles"[MeSH Terms] OR "alleles"[All Fields] OR "allele"[All Fields] OR "genomics"[MeSH Terms] OR "genomics"[All Fields] OR "genetic"[All Fields] OR "exome"[MeSH Terms] OR "exome"[All Fields] OR "base sequence"[MeSH Terms] OR "sequence"[All Fields]) AND ("Disease Susceptibility"[Mesh Terms] OR "susceptibility"[All Fields] OR "susceptibility"[All Fields] OR "sensitivity"[All Fields] OR "risk"[MeSH Terms] OR "risk"[All Fields] OR Severity[All Fields] OR "association"[MeSH Terms] OR "association"[All Fields] OR "mortality"[Subheading] OR "mortality"[All Fields] OR "mortality"[MeSH Terms]) AND ("respiratory tract infections"[MeSH Terms] OR ("respiratory"[All Fields] AND "tract"[All Fields] AND "infections"[All Fields]) OR "respiratory tract infection"[All Fields] OR ("respiratory"[All Fields] AND "infection"[All Fields]) OR "respiratory infection"[All Fields] OR "pneumonia"[MeSH Terms] OR "pneumonia"[All Fields] OR "otitis media"[MeSH Terms] OR ("otitis"[All Fields] AND "media"[All Fields]) OR "otitis media"[All Fields] OR "bronchitis"[MeSH Terms] OR "bronchitis"[All Fields] OR "bronchiolitis"[MeSH Terms] OR "bronchiolitis"[All Fields] OR "common cold"[MeSH Terms] OR ("common"[All Fields] AND "cold"[All Fields]) OR "common cold"[All Fields] OR "pharyngitis"[MeSH Terms] OR "pharyngitis"[All Fields] OR "pleurisy"[MeSH Terms] OR "pleurisy"[All Fields] OR "pleuritis"[All Fields] OR "glottis"[MeSH Terms] OR "glottis"[All Fields] OR "sinusitis"[MeSH Terms] OR "sinusitis"[All Fields] OR "tonsillitis"[MeSH Terms] OR "tonsillitis"[All Fields] OR "pharyngitis"[MeSH Terms] OR "pharyngitis"[All Fields] OR "influenza, human"[MeSH Terms] OR ("influenza"[All Fields] AND "human"[All Fields]) OR "human influenza"[All Fields] OR "influenza"[All Fields] OR "flu"[All Fields] OR "streptococcus pneumoniae"[MeSH Terms] OR ("streptococcus"[All Fields] AND "pneumoniae"[All Fields]) OR "streptococcus pneumoniae"[All Fields] OR "pneumococcus"[All Fields] OR "orthomyxoviridae infections"[MeSH Terms] OR ("orthomyxoviridae"[All Fields] AND "infections"[All Fields]) OR "orthomyxoviridae infections"[All Fields] OR "coronavirus infections"[MeSH Terms] OR "coronavirus"[All Fields] OR "coronavirus infections"[All Fields] OR "severe acute respiratory syndrome"[MeSH Terms] OR ("severe"[All Fields] AND "acute"[All Fields] AND "respiratory"[All Fields] AND "syndrome"[All Fields]) OR "severe acute respiratory syndrome"[All Fields] OR "SARS"[All Fields] OR "respiratory syncytial virus infections"[MeSH Terms] OR ("respiratory"[All Fields] AND "syncytial"[All Fields] AND "virus"[All Fields] AND "infections"[All Fields]) OR "respiratory syncytial virus infections"[All Fields] OR "RSV"[All Fields] OR "rhinovirus"[MeSH Terms] OR "rhinovirus"[All Fields] OR "tuberculosis"[MeSH Terms] OR "tuberculosis"[All Fields] OR "mycobacterium"[MeSH Terms] OR "mycobacterium"[All Fields] OR "haemophilus"[MeSH Terms] OR "haemophilus"[All Fields] OR "hemophilus"[All Fields] OR "legionella"[MeSH Terms] OR "legionella"[All Fields] OR ("chlamydia"[MeSH Terms] OR "chlamydia"[All Fields]) AND ("pneumonia"[MeSH Terms] OR "pneumonia"[All Fields]) OR "paramyxoviridae infections"[MeSH Terms] OR ("paramyxoviridae"[All Fields] AND "infections"[All Fields]) OR "paramyxoviridae infections"[All Fields] OR "parainfluenza"[All Fields] OR "adenoviridae"[MeSH Terms] OR "adenoviridae"[All Fields] OR "adenovirus"[All Fields] OR "adenoviridae infections"[MeSH Terms] OR ("adenoviridae"[All Fields] AND "infections"[All Fields]) OR "adenoviridae infections"[All Fields] OR "mycoplasma pneumoniae"[MeSH Terms] OR ("mycoplasma"[All Fields] AND "pneumoniae"[All Fields]) OR "mycoplasma pneumoniae"[All Fields]) AND ("humans"[MeSH Terms] OR "humans"[All Fields] OR "human"[All Fields]) AND ("2000/01/01"[PDAT] : "3000"[PDAT]) AND English[lang] |
| **Web of Knoledge (wok.mimas.ac.uk)** |
| TOPIC: ((DNA OR SNP OR gene OR variant OR polymorphism OR genotype OR allele OR genetic OR genom* OR exom* OR sequenc*) AND (susceptib* OR sensitiv* OR association OR sever* OR mortality OR risk) AND (respiratory tract infection OR respiratory infection OR Pneumonia OR otitis media OR bronchitis OR bronchiolitis OR common cold OR pharyngitis OR pleurisy OR pleuritis OR glottis OR sinusitis OR tonsillitis OR influenza OR flu OR streptococcus pneumoniae OR pneumococcus OR orthomyxovir* OR coronavir* OR severe acute respiratory syndrome OR SARS OR respiratory syncytial virus OR RSV OR rhinovirus OR tuberculosis OR mycobacterium OR haemophilus OR legionella OR chlamydia pneumonia OR paramyxovir* OR parainfluenza OR adenovir* OR mycoplasma) AND human) AND YEAR PUBLISHED:(2000-2015) AND LANGUAGE:(English) |
| **SCOPUS (http://www.scopus.com/)** |
| TITLE-ABS-KEY(DNA OR SNP OR gene OR variant OR polymorphism OR genotype OR allele OR genetic OR genom* OR exom* OR sequenc*) AND (susceptib* OR sensitiv* OR associate* OR sever* OR mortality OR risk) AND (respiratory tract infection OR Pneumonia OR otitis media OR bronchitis OR bronchiolitis OR common cold OR pharyngitis OR pleurisy OR pleuritis OR glottis OR sinusitis OR tonsillitis OR influenza OR flu OR streptococcus OR pneumococcus OR orthomyxovir* OR coronavir* OR severe acute respiratory syndrome OR SARS OR respiratory syncytial virus OR RSV OR rhinovirus OR tuberculosis OR mycobacterium OR haemophilus OR legionella OR chlamydia pneumonia OR paramyxovir* OR parainfluenza OR adenovir* OR mycoplasma) AND (human) AND PUBYEAR > 1999 AND LANGUAGE(english) |

Records identified through database searching

(n =30,867; PubMed: 4,548; WOK 13,418; SCOPUS 11,521; HuGe 1,380

**Screening**

**Included**

**Eligibility**

**Identification**

Additional records identified through references search

(n = 265)

Records after duplicates removed

(n =24,823 )

Records screened

(n =1,146)

Records excluded

(n =23,677)

Full-text articles assessed for eligibility

(n = 1,146)

Excluded articles (n =730):

Reviews: 143

Wrong marker type: 124

Insufficient information: 124

Not relevant: 105

Wrong phenotype: 98

Systematic review/meta-analyses: 54

Not English: 48

Duplicate data/publication: 14

Not retrievable: 10

Conferencesand editorials: 10

Studies included in qualitative synthesis

(n =416)

Studies included in quantitative synthesis (meta-analysis)

(n =386)

Family-based and linkage studies: 19

GWAS studies: 11

Supplementary Figure 5. PRISMA guidelines study flowchart and data extraction process scheme

Supplementary Table 2. Domains and grades of CSI score

| Domain | Level A grade | Level B grade | Level C grade |
| --- | --- | --- | --- |
| Confounding risk | No apparent confounding (or possible confounding properly adjusted for) AND no indication of population stratification | Some degree of confounding possible/probable OR study performed in obviously admixed population | Detectable levels of confounding OR indication of strong stratification |
| Selection bias risk | Controls drawn from general population AND satisfy Hardy-Weinberg equilibrium (HWE)* | Controls drawn from structured sampling frame (hospital, clinic or health care programme-based) AND in HWE | No description on controls recruitment OR controls fail HWE |
| Information bias risk | I1: Cases: status verified by highly specific molecular methods (antigen test, PCR)  I2: Controls: status verified by highly specific molecular methods (antigen test, PCR)  I3: Genotyping: favourable quality control estimates given, subset or total dataset replicated | I1: Cases status established on the basis of guidelines, clinical status, or less specific methods (isolation, smears and microbiological cultures)  I2: Controls status inferred from medical records only (no history of disease)  I3: Partial genotyping quality control results | I1: No clear case definition provided  I2: No description of disease status in controls (“healthy” controls)  I3: No indication of genotyping reproducibility |

*in several instances authors referred to “random” controls sampling; if sufficient information was provided in regards to sampling frame and/or accounting for non-response, these studies were retained in A score. If such information was lacking, the study score was downgraded to C score.

Supplementary Table 3. Meta-analysis results, allelic model

| Gene | rs code | Heterozygote | Risk allele | N studies | N cases (allelels) | N Controls (allelels) | OR [95% CI] | P | I^2^ [95% CI] | Venice score | BFDP (med/low) | BFDP (very low) |
| --- | --- | --- | --- | --- | --- | --- | --- | --- | --- | --- | --- | --- |
| **Pooled result** |  |  |  |  |  |  |  |  |  |  |  |  |
| ACE | rs4340 | D/I | D | 6 | 2888 | 4226 | 1.05 [0.96-1.16] | 0.297 | 0.00 [0.00-0.74] | - | - | - |
| CCL2 | rs3760396 | CG | C | 4 | 2134 | 1824 | 0.96 [0.68-1.36] | 0.812 | 0.17 [0.00-0.87] | - | - | - |
| CCL2 | rs3917891 | CT | T | 5 | 6112 | 6444 | 1.14 [1.04-1.25] | 0.006 | 0.00 [0.00-0.50] | BAC | 0.680 | 0.991 |
| CCL2 | rs2530797 | [CT] | C | 5 | 3016 | 2800 | 1.08 [0.90-1.28] | 0.403 | 0.32 [0.00-0.74] | - | - | - |
| CCL2 | rs1024610 | AT | T | 6 | 6950 | 7400 | 0.95 [0.82-1.10] | 0.495 | 0.03 [0.00-0.75] | - | - | - |
| CCL2 | rs2857656 | CG | C | 6 | 6532 | 6870 | 0.97 [0.85-1.12] | 0.699 | 0.59 [0.00-0.83] | - | - | - |
| CCL2 | rs4586 | CT | T | 8 | 7764 | 8676 | 0.96 [0.88-1.06] | 0.428 | 0.29 [0.00-0.68] | - | - | - |
| CCL2 | rs1024611 | AG | G | 19 | 16058 | 18458 | 0.90 [0.77-1.04] | 0.155 | 0.88 [0.83-0.92] | - | - | - |
| CCL5 | rs2280789 | CT | G | 8 | 4064 | 4946 | 0.94 [0.81-1.09] | 0.382 | 0.44 [0.00-0.75] | - | - | - |
| CCL5 | rs2280788 | CG | C | 10 | 4582 | 5346 | 0.66 [0.36-1.19] | 0.167 | 0.93 [0.89-0.95] | - | - | - |
| CCL5 | rs2107538 | AG | T | 15 | 8390 | 10326 | 1.07 [0.96-1.20] | 0.233 | 0.63 [0.36-0.79] | - | - | - |
| CCR2 | rs1799864 | AG | A | 4 | 1956 | 3646 | 0.92 [0.69-1.23] | 0.586 | 0.62 [0.00-0.87] | - | - | - |
| CCR5 | rs1799987 | AG | A | 4 | 2614 | 4236 | 0.99 [0.89-1.09] | 0.784 | 0.00 [0.00-0.70] | - | - | - |
| CD14 | rs2569190 | CT | A | 14 | 4386 | 7324 | 0.90 [0.75-1.08] | 0.245 | 0.80 [0.67-0.88] | - | - | - |
| CD209 | rs735239 | AG | G | 5 | 2688 | 2920 | 1.32 [1.03-1.69] | 0.027 | 0.64 [0.04-0.86] | BCC | 0.862 | 0.997 |
| CD209 | rs735240 | AG | A | 6 | 3596 | 5390 | 0.91 [0.81-1.01] | 0.084 | 0.18 [0.00-0.63] | - | - | - |
| CD209 | rs2287886 | AG | A | 6 | 3316 | 4842 | 1.07 [0.92-1.24] | 0.400 | 0.53 [0.00-0.81] | - | - | - |
| CD209 | rs4804803 | AG | G | 11 | 4922 | 6548 | 0.99 [0.85-1.14] | 0.848 | 0.47 [0.00-0.74] | - | - | - |
| FCGR2A | rs1801274 | AG | G | 8 | 3830 | 4918 | 0.94 [0.73-1.21] | 0.646 | 0.81 [0.62-0.90] | - | - | - |
| IFNG | rs1861494 | AG | C | 5 | 4336 | 5056 | 1.04 [0.81-1.34] | 0.740 | 0.80 [0.52-0.91] | - | - | - |
| IFNG | rs2430561 | AT | A | 28 | 10948 | 12304 | 1.30 [1.07-1.58] | 0.009 | 0.90 [0.86-0.92] | ACC | 0.723 | 0.993 |
| IL10 | rs1800871 | CT | A | 16 | 7880 | 11134 | 1.00 [0.93-1.08] | 0.967 | 0.20 [0.00-0.56] | - | - | - |
| IL10 | rs1800872 | AC | T | 21 | 9573 | 15366 | 0.94 [0.83-1.06] | 0.308 | 0.74 [0.60-0.83] | - | - | - |
| IL10 | rs1800896 | AG | C | 31 | 13034 | 18912 | 1.02 [0.93-1.12] | 0.706 | 0.57 [0.36-0.71] | - | - | - |
| IL12B | rs3212227 | AC | G | 10 | 5390 | 7108 | 0.98 [0.90-1.07] | 0.664 | 0.15 [0.00-0.56] | - | - | - |
| IL12RB1 | rs11575934 | AG | C | 4 | 1010 | 1828 | 0.85 [0.64-1.13] | 0.275 | 0.62 [0.00-0.87] | - | - | - |
| IL12RB1 | rs401502 | CG | G | 4 | 1062 | 1876 | 1.32 [0.93-1.87] | 0.123 | 0.48 [0.00-0.83] | - | - | - |
| IL12RB1 | rs375947 | CT | G | 5 | 2108 | 4238 | 1.12 [0.91-1.38] | 0.275 | 0.63 [0.03-0.86] | - | - | - |
| IL13 | rs20541 | AG | A | 4 | 2070 | 5148 | 0.94 [0.76-1.16] | 0.589 | 0.61 [0.00-0.87] | - | - | - |
| IL13 | rs1800925 | CT | C | 4 | 2084 | 5204 | 0.88 [0.76-1.03] | 0.111 | 0.25 [0.00-0.71] | - | - | - |
| IL18 | rs187238 | CG | G | 5 | 2174 | 3548 | 1.00 [0.86-1.15] | 0.958 | 0.00 [0.00-0.24] | - | - | - |
| IL18 | rs1946518 | [AC] | T | 7 | 3536 | 6014 | 1.09 [0.95-1.25] | 0.213 | 0.57 [0.00-0.81] | - | - | - |
| IL1A | rs1800587 | CT | A | 4 | 1230 | 3032 | 0.92 [0.79-1.07] | 0.267 | 0.00 [0.00-0.74] | - | - | - |
| IL1B | rs1143634 | [CT] | A | 8 | 2844 | 4126 | 0.85 [0.68-1.07] | 0.170 | 0.58 [0.08-0.81] | - | - | - |
| IL1B | rs16944 | [CT] | A | 9 | 4044 | 5909 | 0.88 [0.81-0.97] | 0.007 | 0.00 [0.00-0.65] | BAC | 0.781 | 0.995 |
| IL2 | rs2069762 | GT | C | 7 | 2274 | 4800 | 1.12 [0.98-1.27] | 0.107 | 0.26 [0.00-0.68] | - | - | - |
| IL4 | rs2243248 | GT | G | 4 | 1306 | 2410 | 0.74 [0.41-1.34] | 0.318 | 0.86 [0.65-0.94] | - | - | - |
| IL4 | rs2070874 | CT | T | 6 | 2908 | 6422 | 0.79 [0.70-0.89] | 7.68E-05 | 0.06 [0.00-0.76] | BAC | 0.063 | 0.779 |
| IL4 | rs2243250 | CT | T | 13 | 5114 | 10288 | 0.92 [0.79-1.08] | 0.314 | 0.69 [0.46-0.83] | - | - | - |
| IL4RA | rs1801275 | [AG] | G | 5 | 1816 | 4320 | 1.07 [0.93-1.23] | 0.346 | 0.00 [0.00-0.14] | - | - | - |
| IL6 IL6R | rs1800797 | AG | A | 4 | 2034 | 3578 | 0.93 [0.80-1.08] | 0.339 | 0.00 [0.00-0.76] | - | - | - |
| IL6 IL6R | rs1800795 | CG | C | 14 | 9092 | 10188 | 0.90 [0.82-0.99] | 0.030 | 0.33 [0.00-0.64] | BBC | 0.900 | 0.998 |
| IL8 | rs4073 | AT | T | 8 | 3538 | 5886 | 0.96 [0.81-1.13] | 0.588 | 0.68 [0.33-0.85] | - | - | - |
| MBL2 | rs7095891 | CT | A | 6 | 4314 | 8350 | 1.05 [0.96-1.14] | 0.277 | 0.00 [0.00-0.75] | - | - | - |
| MBL2 | rs11003125 | CG | C | 8 | 4758 | 8898 | 1.12 [0.99-1.26] | 0.075 | 0.42 [0.00-0.74] | - | - | - |
| MBL2 | rs1800450 | AG (AB) | T | 10 | 2506 | 4510 | 1.09 [0.81-1.46] | 0.576 | 0.68 [0.38-0.83] | - | - | - |
| MBL2 | rs7096206 | CG | G | 11 | 8014 | 15906 | 1.00 [0.88-1.13] | 0.991 | 0.57 [0.16-0.78] | - | - | - |
| MIF | rs755622 | CG | C | 4 | 4414 | 2546 | 1.33 [0.94-1.88] | 0.111 | 0.85 [0.62-0.94] | - | - | - |
| OAS1 | rs2660 | AG | A | 5 | 14986 | 21024 | 1.01 [0.89-1.14] | 0.923 | 0.70 [0.22-0.88] | - | - | - |
| P2RX7 | rs2393799 | CT | T | 11 | 4930 | 6094 | 0.89 [0.73-1.08] | 0.242 | 0.78 [0.61-0.88] | - | - | - |
| P2RX7 | rs3751143 | AC | C | 13 | 4526 | 5922 | 0.83 [0.70-0.98] | 0.025 | 0.56 [0.18-0.76] | BCC | 0.864 | 0.997 |
| PTPN22 | rs2476601 | CT | A | 6 | 1916 | 3358 | 1.40 [0.71-2.75] | 0.326 | 0.66 [0.18-0.86] | - | - | - |
| SFTPC | rs1124 | [AG] | A | 4 | 1384 | 1850 | 0.96 [0.71-1.32] | 0.816 | 0.54 [0.00-0.85] | - | - | - |
| SLC11A1 | rs17235409 | AG | A | 27 | 7888 | 8634 | 1.20 [1.04-1.39] | 0.012 | 0.52 [0.25-0.69] | BCC | 0.804 | 0.995 |
| SLC11A1 | rs17235416 | +/del | - | 24 | 8820 | 10008 | 0.80 [0.71-0.91] | 0.001 | 0.54 [0.27-0.71] | BCC | 0.251 | 0.946 |
| SLC11A1 | rs3731865 | CG | C | 18 | 5472 | 6372 | 1.39 [1.07-1.79] | 0.012 | 0.75 [0.61-0.84] | BCC | 0.775 | 0.995 |
| SP110 | rs9061 | AG | A | 4 | 2808 | 1903 | 1.06 [0.81-1.38] | 0.676 | 0.60 [0.00-0.87] | - | - | - |
| SP110 | rs11556887 | CT | A | 4 | 5890 | 5335 | 1.07 [0.78-1.48] | 0.666 | 0.72 [0.22-0.90] | - | - | - |
| SP110 | rs1365776 | AG | A | 4 | 5964 | 6133 | 0.99 [0.91-1.07] | 0.763 | 0.00 [0.00-0.82] | - | - | - |
| SP110 | rs1135791 | CT | G | 4 | 3390 | 2595 | 1.00 [0.77-1.29] | 0.986 | 0.76 [0.33-0.91] | - | - | - |
| SP110 | rs3948464 | CT | A | 5 | 6130 | 6255 | 1.05 [0.92-1.20] | 0.445 | 0.06 [0.00-0.81] | - | - | - |
| SP-A1 | rs1059047 | CT | C | 5 | 2454 | 3722 | 0.97 [0.64-1.46] | 0.884 | 0.68 [0.18-0.88] | - | - | - |
| SP-A1 | rs1136450 | CG | G | 5 | 2454 | 3722 | 1.03 [0.85-1.25] | 0.792 | 0.54 [0.00-0.83] | - | - | - |
| SP-A1 | rs4253527 | CT | T | 5 | 2454 | 3722 | 1.07 [0.79-1.44] | 0.684 | 0.57 [0.00-0.84] | - | - | - |
| SP-A2 | rs1059046 | AC | A | 5 | 2454 | 3722 | 0.82 [0.73-0.92] | 0.001 | 0.00 [0.00-0.59] | BAC | 0.260 | 0.949 |
| SP-A2 | rs17886395 | CG | G | 5 | 2454 | 3722 | 0.83 [0.48-1.42] | 0.490 | 0.90 [0.79-0.95] | - | - | - |
| SP-A2 | rs1965708 | AC | T | 5 | 2454 | 3722 | 1.21 [1.01-1.45] | 0.041 | 0.21 [0.00-0.67] | BAC | 0.887 | 0.998 |
| SP-D | rs2243639 | [AG] | A | 4 | 1402 | 4180 | 0.97 [0.75-1.26] | 0.844 | 0.65 [0.00-0.88] | - | - | - |
| SP-D | rs721917 | [CT] | G | 6 | 3754 | 6482 | 0.98 [0.83-1.14] | 0.771 | 0.61 [0.06-0.84] | - | - | - |
| TGFB1 | rs1800471 | CG | G | 4 | 1324 | 3094 | 1.15 [0.90-1.46] | 0.270 | 0.00 [0.00-0.22] | - | - | - |
| TIRAP | rs8177374 | CT | T | 10 | 12966 | 15286 | 1.11 [0.86-1.44] | 0.408 | 0.67 [0.35-0.83] | - | - | - |
| TLR1 | rs5743618 | GT | C | 5 | 2022 | 2746 | 1.14 [0.84-1.55] | 0.390 | 0.47 [0.00-0.80] | - | - | - |
| TLR2 | rs3804099 | CT | C | 7 | 3204 | 3958 | 1.14 [1.03-1.26] | 0.010 | 0.00 [0.00-0.6] | BAC | 0.781 | 0.995 |
| TLR2 | rs5743708 | AG | A | 11 | 5376 | 7586 | 1.26 [0.75-2.11] | 0.386 | 0.63 [0.29-0.81] | - | - | - |
| TLR4 | rs4986791 | [CT] | T | 12 | 5248 | 10948 | 0.81 [0.35-1.88] | 0.624 | 0.96 [0.95-0.97] | - | - | - |
| TLR4 | rs4986790 | AG | G | 19 | 7682 | 24496 | 0.88 [0.53-1.46] | 0.618 | 0.94 [0.92-0.96] | - | - | - |
| TLR5 | rs5744168 | CT | A | 4 | 1724 | 4326 | 0.92 [0.63-1.35] | 0.673 | 0.51 [0.00-0.84] | - | - | - |
| TLR6 | rs5743810 | CT | A | 5 | 1956 | 4116 | 0.86 [0.74-0.99] | 0.040 | 0.20 [0.00-0.66] | BAC | 0.890 | 0.998 |
| TLR9 | rs187084 | [CT] | G | 4 | 1048 | 1326 | 0.94 [0.79-1.11] | 0.458 | 0.00 [0.00-0.78] | - | - | - |
| TNFA | rs1800630 | AC | A | 5 | 1986 | 2510 | 1.01 [0.84-1.21] | 0.923 | 0.13 [0.00-0.82] | - | - | - |
| TNFA | rs1799724 | CT | T | 8 | 3232 | 4498 | 1.07 [0.80-1.43] | 0.632 | 0.73 [0.46-0.87] | - | - | - |
| TNFA | rs361525 | AG | A | 19 | 6878 | 10396 | 1.40 [0.96-2.02] | 0.078 | 0.85 [0.78-0.90] | - | - | - |
| TNFA | rs1800629 | AG | A | 38 | 19710 | 23478 | 1.12 [0.99-1.26] | 0.066 | 0.38 [0.05-0.60] | - | - | - |
| TNFB_LTA | rs909253 | [AG] | G | 5 | 3238 | 3676 | 0.90 [0.70-1.15] | 0.395 | 0.66 [0.12-0.87] | - | - | - |
| VDR | rs7975232 | Aa | A | 11 | 2660 | 3516 | 1.10 [0.96-1.26] | 0.172 | 0.27 [0.00-0.64] | - | - | - |
| VDR | rs1544410 | Bb | T | 15 | 4616 | 6130 | 1.02 [0.85-1.23] | 0.815 | 0.73 [0.55-0.84] | - | - | - |
| VDR | rs731236 | Tt | G | 23 | 7242 | 9806 | 0.89 [0.78-1.02] | 0.090 | 0.60 [0.37-0.75] | - | - | - |
| VDR | rs2228570 | Ff | A | 23 | 5304 | 6096 | 0.94 [0.81-1.10] | 0.428 | 0.65 [0.45-0.77] | - | - | - |
| **Tuberculosis** |  |  |  |  |  |  |  |  |  |  |  |  |
| CCL2 | rs1024611 | AG | G | 18 | 15244 | 16442 | 0.89 [0.76-1.05] | 0.157 | 0.89 [0.84-0.92] | - | - | - |
| CCL2 | rs4586 | CT | T | 8 | 7764 | 8676 | 0.96 [0.88-1.06] | 0.428 | 0.29 [0.00-0.68] | - | - | - |
| CCL2 | rs3917891 | CT | T | 5 | 6112 | 6444 | 1.14 [1.04-1.25] | 0.006 | 0.00 [0.00-0.50] | BAC | 0.680 | 0.991 |
| CCL5 | rs2107538 | AG | T | 9 | 4904 | 5234 | 1.14 [0.97-1.35] | 0.121 | 0.71 [0.43-0.85] | - | - | - |
| CCL5 | rs2280788 | CG | C | 6 | 2550 | 2814 | 0.55 [0.20-1.54] | 0.256 | 0.94 [0.90-0.97] | - | - | - |
| CD14 | rs2569190 | CT | A | 6 | 2144 | 2654 | 0.80 [0.55-1.16] | 0.245 | 0.89 [0.79-0.94] | - | - | - |
| CD209 | rs2287886 | AG | A | 4 | 2164 | 2486 | 1.08 [0.85-1.37] | 0.537 | 0.67 [0.05-0.89] | - | - | - |
| CD209 | rs4804803 | AG | G | 9 | 3746 | 4192 | 1.01 [0.84-1.20] | 0.953 | 0.55 [0.06-0.79] | - | - | - |
| CD209 | rs735239 | AG | G | 5 | 2688 | 2920 | 1.32 [1.03-1.69] | 0.027 | 0.64 [0.04-0.86] | BCC | 0.862 | 0.997 |
| IFNG | rs2430561 | AT | A | 26 | 9802 | 10572 | 1.28 [1.05-1.56] | 0.016 | 0.89 [0.85-0.92] | BCC | 0.795 | 0.995 |
| IFNG | rs1861494 | AG | C | 5 | 4336 | 5056 | 1.04 [0.81-1.34] | 0.740 | 0.80 [0.52-0.91] | - | - | - |
| IL10 | rs1800872 | AC | T | 15 | 6925 | 10610 | 0.95 [0.82-1.10] | 0.505 | 0.73 [0.55-0.84] | - | - | - |
| IL10 | rs1800896 | AG | C | 25 | 10364 | 14258 | 1.00 [0.90-1.11] | 0.936 | 0.55 [0.29-0.71] | - | - | - |
| IL10 | rs1800871 | CT | A | 15 | 7750 | 11042 | 1.00 [0.92-1.07] | 0.898 | 0.16 [0.00-0.53] | - | - | - |
| IL12B | rs3212227 | AC | G | 9 | 4576 | 5092 | 1.00 [0.91-1.10] | 0.987 | 0.14 [0.00-0.56] | - | - | - |
| IL18 | rs1946518 | AC | T | 5 | 2534 | 3810 | 1.10 [0.91-1.34] | 0.314 | 0.68 [0.17-0.88] | - | - | - |
| IL1B | rs1143634 | [CT] | A | 7 | 2030 | 2110 | 0.83 [0.63-1.09] | 0.179 | 0.57 [0.00-0.81] | - | - | - |
| IL1B | rs16944 | [CT] | A | 7 | 3130 | 3541 | 0.87 [0.78-0.98] | 0.022 | 0.08 [0.00-0.73] | BAC | 0.857 | 0.997 |
| IL2 | rs2069762 | GT | C | 6 | 1460 | 2784 | 1.16 [0.98-1.36] | 0.079 | 0.27 [0.00-0.70] | - | - | - |
| IL4 | rs2243250 | CT | T | 7 | 2268 | 3632 | 1.06 [0.85-1.32] | 0.611 | 0.67 [0.26-0.85] | - | - | - |
| IL6 IL6R | rs1800795 | CG | C | 6 | 2024 | 2524 | 0.78 [0.63-0.96] | 0.019 | 0.14 [0.00-0.78] | BAC | 0.825 | 0.996 |
| MBL2 | rs1800450 | AG (AB) | T | 6 | 1290 | 1950 | 1.06 [0.70-1.60] | 0.801 | 0.66 [0.18-0.86] | - | - | - |
| MBL2 | rs7096206 | CG (XY) | G | 5 | 3178 | 6158 | 1.02 [0.80-1.29] | 0.883 | 0.63 [0.01-0.86] | - | - | - |
| P2RX7 | rs3751143 | AC | C | 13 | 4526 | 5922 | 0.83 [0.70-0.98] | 0.025 | 0.56 [0.18-0.76] | BCC | 0.864 | 0.997 |
| P2RX7 | rs2393799 | CT | T | 11 | 4930 | 6094 | 0.89 [0.73-1.08] | 0.242 | 0.78 [0.61-0.88] | - | - | - |
| PTPN22 | rs2476601 | CT | A | 5 | 1694 | 1752 | 1.74 [0.92-3.30] | 0.089 | 0.35 [0.00-0.75] | - | - | - |
| SLC11A1 | rs17235409 | AG | A | 27 | 7888 | 8634 | 1.20 [1.04-1.39] | 0.012 | 0.52 [0.25-0.69] | BCC | 0.804 | 0.995 |
| SLC11A1 | rs17235416 | +/del | - | 24 | 8820 | 10008 | 0.80 [0.71-0.91] | 0.001 | 0.54 [0.27-0.71] | BCC | 0.251 | 0.946 |
| SLC11A1 | rs3731865 | CG | C | 18 | 5472 | 6372 | 1.39 [1.07-1.79] | 0.012 | 0.75 [0.61-0.84] | BCC | 0.775 | 0.995 |
| SP110 | rs3948464 | CT | A | 5 | 6130 | 6255 | 1.05 [0.92-1.20] | 0.445 | 0.06 [0.00-0.81] | - | - | - |
| SP110 | rs9061 | AG | T | 4 | 2808 | 1903 | 1.06 [0.81-1.38] | 0.676 | 0.60 [0.00-0.87] | - | - | - |
| SP110 | rs11556887 | CT | A | 4 | 5890 | 5335 | 1.07 [0.78-1.48] | 0.666 | 0.72 [0.22-0.90] | - | - | - |
| SP110 | rs1365776 | AG | C | 4 | 5964 | 6133 | 0.99 [0.91-1.07] | 0.763 | 0.00 [0.00-0.82] | - | - | - |
| SP110 | rs1135791 | CT | G | 4 | 3390 | 2595 | 1.00 [0.77-1.29] | 0.986 | 0.76 [0.33-0.91] | - | - | - |
| TIRAP | rs8177374 | CT | T | 10 | 12966 | 15286 | 1.11 [0.86-1.44] | 0.408 | 0.67 [0.35-0.83] | - | - | - |
| TLR2 | rs5743708 | AG | A | 5 | 3262 | 3124 | 3.19 [2.03-5.02] | 4.91E-07 | 0.00 [0.00-0.63] | BAC | 0.087 | 0.834 |
| TLR2 | rs3804099 | CT | C | 5 | 2722 | 2898 | 1.14 [1.01-1.27] | 0.028 | 0.00 [0.00-0.78] | BAC | 0.840 | 0.996 |
| TLR4 | rs4986791 | CT | T | 4 | 2316 | 2158 | 0.93 [0.68-1.27] | 0.639 | 0.33 [0.00-0.76] | - | - | - |
| TLR4 | rs4986790 | AG | G | 7 | 3478 | 3966 | 0.80 [0.62-1.04] | 0.091 | 0.38 [0.00-0.74] | - | - | - |
| TNFA | rs1799724 | CT | T | 5 | 1966 | 1680 | 0.94 [0.57-1.55] | 0.808 | 0.84 [0.65-0.93] | - | - | - |
| TNFA | rs361525 | AG | A | 12 | 2796 | 4478 | 1.35 [0.76-2.41] | 0.307 | 0.89 [0.82-0.93] | - | - | - |
| TNFA | rs1800629 | AG | A | 20 | 5334 | 7714 | 1.11 [0.95-1.31] | 0.190 | 0.32 [0.00-0.60] | - | - | - |
| TNFA | rs1800630 | AC | A | 4 | 1836 | 2248 | 0.98 [0.78-1.25] | 0.898 | 0.33 [0.00-0.76] | - | - | - |
| VDR | rs1544410 | Bb | T | 14 | 3802 | 4114 | 1.03 [0.83-1.28] | 0.767 | 0.75 [0.58-0.85] | - | - | - |
| VDR | rs731236 | Tt | G | 20 | 6124 | 7456 | 0.88 [0.75-1.04] | 0.134 | 0.65 [0.44-0.78] | - | - | - |
| VDR | rs2228570 | Ff | A | 21 | 4990 | 5766 | 0.96 [0.82-1.13] | 0.634 | 0.66 [0.45-0.78] | - | - | - |
| VDR | rs7975232 | Aa | A | 10 | 2468 | 3314 | 1.12 [0.97-1.29] | 0.132 | 0.31 [0.00-0.67] | - | - | - |
| **Influenza** |  |  |  |  |  |  |  |  |  |  |  |  |
| There were no SNPs with 4 or more studies used for meta-analysis | | | | | | | | | | | | |
| **Respiratory syncytial virus** | |  |  |  |  |  |  |  |  |  |  |  |
| CCL5 | rs2107538 | [AG] | T | 4 | 1784 | 3202 | 0.95 [0.73-1.23] | 0.683 | 0.65 [0.00-0.88] | - | - | - |
| CD14 | rs2569190 | CT | A | 5 | 1628 | 3316 | 0.96 [0.80-1.14] | 0.605 | 0.34 [0.00-0.75] | - | - | - |
| IL13 | rs20541 | AG | A | 4 | 2070 | 5148 | 0.94 [0.76-1.16] | 0.589 | 0.61 [0.00-0.87] | - | - | - |
| IL13 | rs1800925 | CT | C | 4 | 2084 | 5204 | 0.88 [0.76-1.03] | 0.111 | 0.25 [0.00-0.71] | - | - | - |
| IL4 | rs2243250 | CT | T | 6 | 2846 | 6656 | 0.80 [0.68-0.95] | 0.010 | 0.49 [0.00-0.80] | BBC | 0.758 | 0.994 |
| IL8 | rs4073 | AT | T | 4 | 2386 | 3874 | 0.92 [0.68-1.24] | 0.577 | 0.85 [0.63-0.94] | - | - | - |
| TLR4 | rs4986791 | [CT] | T | 5 | 2078 | 7504 | 0.46 [0.08-2.71] | 0.392 | 0.98 [0.97-0.99] | - | - | - |
| TLR4 | rs4986790 | [AG] | G | 9 | 3350 | 19244 | 0.85 [0.32-2.23] | 0.735 | 0.97 [0.96-0.98] | - | - | - |
| **SARS-Coronavirus** | |  |  |  |  |  |  |  |  |  |  |  |
| There were no SNPs with 4 or more studies used for meta-analysis | | | | | | | | | | | | |
| **Pneumonia** |  |  |  |  |  |  |  |  |  |  |  |  |
| FCGR2A | rs1801274 | AG | G | 6 | 3408 | 4422 | 0.82 [0.65-1.05] | 0.110 | 0.72 [0.35-0.88] | - | - | - |
| IL6 IL6R | rs1800795 | CG | C | 6 | 6124 | 5556 | 0.95 [0.84-1.06] | 0.348 | 0.40 [0.00-0.76] | - | - | - |

Supplementary Table 4. Meta-analysis results, dominant model

| Gene | | rs code | Heterozygote | Risk allele | N studies | N cases (allelels) | N Controls (allelels) | OR [95% CI] | P | I^2^ [95% CI] | Venice score | BFDP (med/low) | BFDP (very low) |
| --- | --- | --- | --- | --- | --- | --- | --- | --- | --- | --- | --- | --- | --- |
| **Pooled result** | |  |  |  |  |  |  |  |  |  |  |  |  |
| ACE | | rs4340 | D/I | D | 6 | 1444 | 2113 | 1.14 [0.92-1.41] | 0.223 | 0.43 [0.00-0.78] | - | - | - |
| CCL2 | | rs3917891 | CT | T | 5 | 3056 | 3222 | 1.18 [1.06-1.32] | 0.002 | 0.00 [0.00-0.62] | BAC | 0.593 | 0.987 |
| CCL2 | | rs2530797 | [CT] | C | 5 | 1508 | 1400 | 1.00 [0.56-1.80] | 0.997 | 0.54 [0.00-0.83] | - | - | - |
| CCL2 | | rs1024610 | AT | T | 6 | 3475 | 3700 | 1.00 [0.85-1.18] | 0.984 | 0.00 [0.00-0.71] | - | - | - |
| CCL2 | | rs2857656 | CG | C | 6 | 3266 | 3435 | 0.96 [0.80-1.14] | 0.610 | 0.32 [0.00-0.72] | - | - | - |
| CCL2 | | rs4586 | CT | T | 8 | 3882 | 4338 | 0.90 [0.82-0.99] | 0.026 | 0.00 [0.00-0.62] | BAC | 0.900 | 0.998 |
| CCL2 | | rs1024611 | AG | G | 18 | 7798 | 9078 | 0.88 [0.75-1.04] | 0.149 | 0.82 [0.73-0.88] | - | - | - |
| CCL5 | | rs2280789 | CT | G | 8 | 2032 | 2473 | 1.01 [0.76-1.34] | 0.939 | 0.22 [0.00-0.64] | - | - | - |
| CCL5 | | rs2280788 | CG | C | 10 | 2291 | 2673 | 0.66 [0.35-1.24] | 0.197 | 0.91 [0.85-0.94] | - | - | - |
| CCL5 | | rs2107538 | [AG] | T | 14 | 3760 | 4653 | 1.21 [0.97-1.52] | 0.090 | 0.56 [0.20-0.76] | - | - | - |
| CCR2 | | rs1799864 | AG | A | 4 | 978 | 1823 | 0.98 [0.45-2.13] | 0.957 | 0.49 [0.00-0.83] | - | - | - |
| CCR5 | | rs1799987 | AG | A | 4 | 1307 | 2118 | 0.99 [0.84-1.16] | 0.890 | 0.00 [0.00-0.33] | - | - | - |
| CD14 | | rs2569190 | CT | A | 14 | 2193 | 3662 | 0.93 [0.74-1.17] | 0.542 | 0.66 [0.41-0.81] | - | - | - |
| CD209 | | rs735239 | AG | G | 4 | 1206 | 1320 | 1.64 [1.13-2.38] | 0.010 | 0.74 [0.28-0.91] | BCC | 0.817 | 0.996 |
| CD209 | | rs735240 | AG | A | 5 | 1660 | 2555 | 0.86 [0.69-1.08] | 0.196 | 0.00 [0.00-0.49] | - | - | - |
| CD209 | | rs2287886 | AG | A | 6 | 1658 | 2421 | 1.15 [0.98-1.34] | 0.091 | 0.00 [0.00-0.73] | - | - | - |
| CD209 | | rs4804803 | AG | G | 10 | 2323 | 3134 | 0.97 [0.79-1.20] | 0.808 | 0.62 [0.24-0.81] | - | - | - |
| FCGR2A | | rs1801274 | AG | G | 8 | 1915 | 2459 | 1.12 [0.84-1.49] | 0.451 | 0.60 [0.13-0.82] | - | - | - |
| IFNG | | rs1861494 | AG | C | 5 | 2168 | 2528 | 0.97 [0.76-1.24] | 0.788 | 0.63 [0.01-0.86] | - | - | - |
| IFNG | | rs2430561 | AT | A | 24 | 4285 | 5072 | 1.28 [1.09-1.52] | 0.003 | 0.60 [0.38-0.75] | BCC | 0.628 | 0.989 |
| IL10 | | rs1800871 | CT | A | 14 | 3645 | 5249 | 0.91 [0.74-1.11] | 0.353 | 0.57 [0.22-0.76] | - | - | - |
| IL10 | | rs1800872 | AC | T | 19 | 4170 | 7099 | 1.02 [0.81-1.28] | 0.869 | 0.65 [0.44-0.79] | - | - | - |
| IL10 | | rs1800896 | AG | C | 27 | 5662 | 8596 | 1.09 [0.94-1.27] | 0.246 | 0.60 [0.40-0.74] | - | - | - |
| IL12B | | rs3212227 | AC | G | 9 | 2535 | 3371 | 0.98 [0.87-1.10] | 0.720 | 0.08 [0.00-0.67] | - | - | - |
| IL12RB1 | | rs375947 | [CT] | G | 4 | 1022 | 2025 | 1.04 [0.72-1.51] | 0.826 | 0.63 [0.00-0.88] | - | - | - |
| IL18 | | rs187238 | CG | G | 5 | 1087 | 1774 | 1.02 [0.79-1.32] | 0.862 | 0.00 [0.00-0.73] | - | - | - |
| IL18 | | rs1946518 | [AC] | T | 7 | 1768 | 3007 | 1.18 [0.96-1.43] | 0.112 | 0.38 [0.00-0.74] | - | - | - |
| IL1A | | rs1800587 | CT | A | 4 | 615 | 1516 | 0.89 [0.73-1.08] | 0.241 | 0.00 [0.00-0.00] | - | - | - |
| IL1B | | rs1143634 | [CT] | A | 7 | 1361 | 2016 | 0.84 [0.70-1.00] | 0.052 | 0.00 [0.00-0.00] | - | - | - |
| IL1B | | rs16944 | [CT] | A | 8 | 1966 | 2909 | 0.88 [0.73-1.06] | 0.177 | 0.21 [0.00-0.63] | - | - | - |
| IL2 | | rs2069762 | GT | C | 6 | 973 | 2213 | 1.15 [0.85-1.54] | 0.363 | 0.12 [0.00-0.78] | - | - | - |
| IL4 | | rs2070874 | CT | T | 5 | 1059 | 2210 | 0.87 [0.73-1.04] | 0.118 | 0.03 [0.00-0.80] | - | - | - |
| IL4 | | rs2243250 | CT | T | 11 | 2030 | 3870 | 0.98 [0.76-1.27] | 0.908 | 0.68 [0.40-0.83] | - | - | - |
| IL4RA | | rs1801275 | [AG] | G | 5 | 908 | 2160 | 1.05 [0.89-1.24] | 0.550 | 0.00 [0.00-0.38] | - | - | - |
| IL6 IL6R | | rs1800795 | CG | C | 10 | 4246 | 4776 | 1.00 [0.87-1.15] | 0.976 | 0.01 [0.00-0.63] | - | - | - |
| IL8 | | rs4073 | AT | T | 8 | 1769 | 2943 | 0.95 [0.78-1.17] | 0.651 | 0.47 [0.00-0.76] | - | - | - |
| MLB2 | | rs7095891 | CT | A | 6 | 2157 | 4175 | 1.14 [1.00-1.30] | 0.045 | 0.00 [0.00-0.70] | BAC | 0.916 | 0.998 |
| MLB2 | | rs11003125 | CG | C | 8 | 2379 | 4449 | 1.13 [0.95-1.35] | 0.177 | 0.40 [0.00-0.73] | - | - | - |
| MLB2 | | rs1800450 | AG (AB) | T | 9 | 1230 | 2231 | 0.85 [0.57-1.28] | 0.448 | 0.13 [0.00-0.55] | - | - | - |
| MLB2 | | rs7096206 | CG | G | 9 | 2590 | 4799 | 1.13 [0.94-1.37] | 0.188 | 0.02 [0.00-0.66] | - | - | - |
| MIF | | rs755622 | CG | C | 4 | 2207 | 1273 | 1.64 [0.90-3.00] | 0.109 | 0.68 [0.07-0.89] | - | - | - |
| OAS1 | | rs2660 | AG | A | 5 | 7493 | 10512 | 1.03 [0.84-1.27] | 0.769 | 0.69 [0.20-0.88] | - | - | - |
| P2RX7 | | rs2393799 | CT | T | 11 | 2465 | 3047 | 0.87 [0.67-1.12] | 0.269 | 0.65 [0.33-0.81] | - | - | - |
| P2RX7 | | rs3751143 | AC | C | 13 | 2263 | 2961 | 0.77 [0.63-0.95] | 0.012 | 0.58 [0.21-0.77] | BCC | 0.798 | 0.995 |
| PTPN22 | | rs2476601 | CT | A | 4 | 604 | 1373 | 1.30 [0.58-2.94] | 0.522 | 0.68 [0.07-0.89] | - | - | - |
| SFTPC | | rs1124 | [AG] | A | 4 | 692 | 925 | 0.91 [0.58-1.44] | 0.697 | 0.32 [0.00-0.76] | - | - | - |
| SLC11A1 | | rs17235409 | AG | A | 22 | 3891 | 4278 | 1.18 [0.85-1.65] | 0.330 | 0.00 [0.00-0.45] | - | - | - |
| SLC11A1 | | rs17235416 | +/del | - | 23 | 4357 | 4965 | 0.78 [0.68-0.90] | 0.001 | 0.48 [0.15-0.68] | BBC | 0.249 | 0.946 |
| SLC11A1 | | rs3731865 | CG | C | 11 | 2472 | 2787 | 2.64 [1.37-5.12] | 0.004 | 0.46 [0.00-0.73] | BBC | 0.879 | 0.997 |
| SP-A1 | | rs1059047 | CT | C | 4 | 1141 | 1766 | 1.79 [0.56-5.75] | 0.325 | 0.17 [0.00-0.87] | - | - | - |
| SP-A1 | | rs1136450 | CG | G | 4 | 1141 | 1766 | 1.03 [0.79-1.34] | 0.840 | 0.30 [0.00-0.74] | - | - | - |
| SP-A1 | | rs4253527 | CT | T | 4 | 1141 | 1766 | 0.96 [0.69-1.33] | 0.792 | 0.52 [0.00-0.84] | - | - | - |
| SP-A2 | | rs1059046 | AC | A | 4 | 1141 | 1766 | 0.80 [0.68-0.95] | 0.009 | 0.00 [0.00-0.72] | BAC | 0.758 | 0.994 |
| SP-A2 | | rs17886395 | CG | G | 4 | 1141 | 1766 | 1.02 [0.34-3.11] | 0.966 | 0.85 [0.64-0.94] | - | - | - |
| SP-A2 | | rs1965708 | AC | T | 4 | 1141 | 1766 | 1.15 [0.59-2.26] | 0.675 | 0.41 [0.00-0.80] | - | - | - |
| SP-D | | rs721917 | CT | G | 5 | 1793 | 3148 | 1.09 [0.92-1.28] | 0.322 | 0.00 [0.00-0.00] | - | - | - |
| TIRAP | | rs8177374 | CT | T | 9 | 6483 | 7643 | 1.21 [0.87-1.67] | 0.256 | 0.72 [0.44-0.86] | - | - | - |
| TLR1 | | rs5743618 | GT | C | 5 | 1011 | 1373 | 1.31 [0.86-2.01] | 0.205 | 0.52 [0.00-0.82] | - | - | - |
| TLR2 | | rs3804099 | CT | C | 7 | 1602 | 1979 | 1.36 [1.13-1.64] | 0.001 | 0.00 [0.00-0.61] | BAC | 0.388 | 0.971 |
| TLR4 | | rs4986791 | [CT] | T | 12 | 2599 | 5499 | 0.63 [0.26-1.54] | 0.313 | 0.95 [0.92-0.96] | - | - | - |
| TLR4 | | rs4986790 | AG | G | 17 | 3504 | 11917 | 0.80 [0.46-1.39] | 0.427 | 0.91 [0.88-0.94] | - | - | - |
| TLR5 | | rs5744168 | CT | A | 4 | 862 | 2163 | 0.92 [0.58-1.46] | 0.723 | 0.62 [0.00-0.87] | - | - | - |
| TLR6 | | rs5743810 | CT | A | 4 | 978 | 2058 | 0.89 [0.71-1.13] | 0.347 | 0.24 [0.00-0.88] | - | - | - |
| TLR9 | | rs187084 | [CT] | G | 4 | 499 | 688 | 0.90 [0.63-1.29] | 0.564 | 0.00 [0.00-0.82] | - | - | - |
| TNFA | | rs1800630 | AC | A | 5 | 993 | 1255 | 1.99 [0.97-4.07] | 0.061 | 0.13 [0.00-0.82] | - | - | - |
| TNFA | | rs1799724 | CT | T | 7 | 1465 | 1979 | 0.95 [0.64-1.41] | 0.795 | 0.79 [0.56-0.90] | - | - | - |
| TNFA | | rs361525 | AG | A | 12 | 3439 | 5198 | 1.90 [0.99-3.62] | 0.052 | 0.44 [0.00-0.71] | - | - | - |
| TNFA | | rs1800629 | AG | A | 31 | 8818 | 10885 | 1.16 [0.89-1.51] | 0.263 | 0.00 [0.00-0.00] | - | - | - |
| TNFB_LTA | | rs909253 | [AG] | G | 4 | 1468 | 1568 | 0.65 [0.39-1.09] | 0.100 | 0.80 [0.48-0.93] | - | - | - |
| VDR | | rs7975232 | Aa | A | 9 | 1005 | 1495 | 1.06 [0.86-1.31] | 0.561 | 0.22 [0.00-0.63] | - | - | - |
| VDR | | rs1544410 | Bb | T | 13 | 1983 | 2802 | 0.82 [0.60-1.12] | 0.204 | 0.67 [0.41-0.82] | - | - | - |
| VDR | | rs731236 | Tt | G | 21 | 3296 | 4640 | 0.87 [0.74-1.03] | 0.105 | 0.51 [0.20-0.71] | - | - | - |
| VDR | | rs2228570 | Ff | A | 21 | 2327 | 2785 | 1.00 [0.80-1.25] | 0.993 | 0.67 [0.48-0.79] | - | - | - |
| **Tuberculosis** | |  |  |  |  |  |  |  |  |  |  |  |  |
| CCL2 | | rs3917891 | CT | T | 5 | 3056 | 3222 | 1.18 [1.06-1.32] | 0.002 | 0.00 [0.00-0.62] | BAC | 0.593 | 0.987 |
| CCL2 | | rs4586 | CT | T | 8 | 3882 | 4338 | 0.90 [0.82-0.99] | 0.026 | 0.00 [0.00-0.62] | BAC | 0.900 | 0.998 |
| CCL2 | | rs1024611 | AG | G | 17 | 7391 | 8070 | 0.87 [0.73-1.04] | 0.133 | 0.83 [0.74-0.89] | - | - | - |
| CCL5 | | rs2280788 | CG | C | 6 | 1275 | 1407 | 0.55 [0.18-1.64] | 0.283 | 0.91 [0.84-0.95] | - | - | - |
| CCL5 | | rs2107538 | AG | T | 8 | 2017 | 2107 | 1.49 [1.06-2.10] | 0.021 | 0.67 [0.29-0.84] | BCC | 0.866 | 0.997 |
| CD14 | | rs2569190 | CT | A | 6 | 1072 | 1327 | 0.78 [0.48-1.28] | 0.326 | 0.83 [0.64-0.92] | - | - | - |
| CD209 | | rs2287886 | AG | A | 4 | 1082 | 1243 | 1.13 [0.91-1.41] | 0.272 | 0.07 [0.00-0.86] | - | - | - |
| CD209 | | rs735239 | AG | G | 4 | 1206 | 1320 | 1.64 [1.13-2.38] | 0.010 | 0.74 [0.28-0.91] | BCC | 0.817 | 0.996 |
| CD209 | | rs4804803 | AG | G | 8 | 1735 | 1956 | 1.01 [0.77-1.33] | 0.945 | 0.69 [0.35-0.85] | - | - | - |
| IFNG | | rs1861494 | AG | C | 5 | 2168 | 2528 | 0.97 [0.76-1.24] | 0.788 | 0.63 [0.01-0.86] | - | - | - |
| IFNG | | rs2430561 | AT | A | 23 | 4188 | 4655 | 1.31 [1.11-1.55] | 0.001 | 0.60 [0.36-0.75] | BCC | 0.421 | 0.975 |
| IL10 | | rs1800871 | CT | A | 13 | 3580 | 5203 | 0.90 [0.74-1.10] | 0.315 | 0.59 [0.24-0.78] | - | - | - |
| IL10 | | rs1800872 | AC | T | 14 | 3322 | 5170 | 0.94 [0.74-1.20] | 0.631 | 0.63 [0.35-0.79] | - | - | - |
| IL10 | | rs1800896 | AG | C | 23 | 4893 | 6811 | 1.04 [0.89-1.21] | 0.600 | 0.50 [0.20-0.69] | - | - | - |
| IL12B | | rs3212227 | AC | G | 8 | 2128 | 2363 | 1.00 [0.88-1.15] | 0.952 | 0.10 [0.00-0.71] | - | - | - |
| IL18 | | rs1946518 | AC | T | 5 | 1267 | 1905 | 1.21 [0.91-1.61] | 0.191 | 0.56 [0.00-0.84] | - | - | - |
| IL1B | | rs1143634 | [CT] | A | 6 | 954 | 1008 | 0.76 [0.58-0.99] | 0.040 | 0.00 [0.00-0.00] | CAC | 0.891 | 0.998 |
| IL1B | | rs16944 | [CT] | A | 6 | 1509 | 1725 | 0.87 [0.71-1.06] | 0.159 | 0.00 [0.00-0.67] | - | - | - |
| IL2 | | rs2069762 | GT | C | 5 | 566 | 1205 | 0.98 [0.69-1.39] | 0.915 | 0.00 [0.00-0.75] | - | - | - |
| IL4 | | rs2243250 | CT | T | 7 | 1134 | 1816 | 1.13 [0.78-1.63] | 0.521 | 0.69 [0.31-0.86] | - | - | - |
| MBL2 | | rs1800450 | AG (AB) | T | 5 | 622 | 951 | 0.85 [0.46-1.54] | 0.583 | 0.40 [0.00-0.78] | - | - | - |
| MBL2 | | rs7096206 | CG (XY) | G | 5 | 1589 | 3079 | 0.94 [0.70-1.26] | 0.675 | 0.00 [0.00-0.57] | - | - | - |
| P2RX7 | | rs2393799 | CT | T | 11 | 2465 | 3047 | 0.87 [0.67-1.12] | 0.269 | 0.65 [0.33-0.81] | - | - | - |
| P2RX7 | | rs3751143 | AC | C | 13 | 2263 | 2961 | 0.77 [0.63-0.95] | 0.012 | 0.58 [0.21-0.77] | BCC | 0.798 | 0.995 |
| SLC11A1 | | rs17235409 | AG | A | 22 | 3891 | 4278 | 1.18 [0.85-1.65] | 0.330 | 0.00 [0.00-0.45] | - | - | - |
| SLC11A1 | | rs17235416 | +/del | - | 23 | 4357 | 4965 | 0.78 [0.68-0.90] | 0.001 | 0.48 [0.15-0.68] | BBC | 0.249 | 0.946 |
| SLC11A1 | | rs3731865 | CG | C | 11 | 2472 | 2787 | 2.64 [1.37-5.12] | 0.004 | 0.46 [0.00-0.73] | BBC | 0.879 | 0.997 |
| TIRAP | | rs8177374 | CT | T | 9 | 6483 | 7643 | 1.21 [0.87-1.67] | 0.256 | 0.72 [0.44-0.86] | - | - | - |
| TLR2 | | rs3804099 | CT | C | 5 | 1361 | 1449 | 1.32 [1.06-1.65] | 0.012 | 0.00 [0.00-0.75] | BAC | 0.801 | 0.995 |
| TLR4 | | rs4986791 | CT | T | 4 | 1133 | 1104 | 0.81 [0.59-1.11] | 0.187 | 0.00 [0.00-0.67] | - | - | - |
| TLR4 | | rs4986790 | AG | G | 7 | 1714 | 2008 | 0.83 [0.64-1.08] | 0.172 | 0.17 [0.00-0.61] | - | - | - |
| TNFA | | rs1800630 | AC | A | 4 | 918 | 1124 | 1.77 [0.72-4.37] | 0.212 | 0.30 [0.00-0.74] | - | - | - |
| TNFA | | rs1799724 | CT | T | 5 | 983 | 840 | 0.84 [0.45-1.54] | 0.568 | 0.85 [0.67-0.93] | - | - | - |
| TNFA | | rs361525 | AG | A | 7 | 1398 | 2239 | 1.74 [0.66-4.62] | 0.265 | 0.66 [0.24-0.85] | - | - | - |
| TNFA | | rs1800629 | AG | A | 17 | 2527 | 3722 | 1.47 [0.81-2.68] | 0.202 | 0.00 [0.00-0.00] | - | - | - |
| VDR | | rs7975232 | Aa | A | 8 | 909 | 1394 | 1.08 [0.86-1.36] | 0.516 | 0.30 [0.00-0.69] | - | - | - |
| VDR | | rs1544410 | Bb | T | 12 | 1576 | 1794 | 0.81 [0.55-1.19] | 0.275 | 0.69 [0.44-0.83] | - | - | - |
| VDR | | rs731236 | Tt | G | 18 | 2737 | 3465 | 0.86 [0.70-1.05] | 0.139 | 0.58 [0.30-0.75] | - | - | - |
| VDR | | rs2228570 | Ff | A | 19 | 2170 | 2620 | 1.04 [0.81-1.32] | 0.776 | 0.69 [0.51-0.81] | - | - | - |
| **Influenza** | |  |  |  |  |  |  |  |  |  |  |  |  |
| There were no SNPs with 4 or more studies used for meta-analysis | | | | | | | | | | | | | |
| **Respiratory syncytial virus** | | |  |  |  |  |  |  |  |  |  |  |  |
| CCL5 | rs2107538 | | [AG] | T | 4 | 892 | 1601 | 0.87 [0.62-1.22] | 0.411 | 0.00 [0.00-0.74] | - | - | - |
| CD14 | rs2569190 | | CT | A | 5 | 814 | 1658 | 1.03 [0.85-1.25] | 0.751 | 0.00 [0.00-0.23] | - | - | - |
| IL4 | rs2243250 | | CT | T | 4 | 896 | 2054 | 0.82 [0.61-1.09] | 0.170 | 0.49 [0.00-0.83] | - | - | - |
| IL8 | rs4073 | | AT | T | 4 | 1193 | 1937 | 0.93 [0.63-1.36] | 0.709 | 0.74 [0.28-0.91] | - | - | - |
| TLR4 | rs4986791 | | [CT] | T | 5 | 1039 | 3752 | 0.34 [0.04-2.62] | 0.300 | 0.98 [0.97-0.99] | - | - | - |
| TLR4 | rs4986790 | | [AG] | G | 7 | 1363 | 9266 | 0.63 [0.17-2.31] | 0.487 | 0.96 [0.95-0.98] | - | - | - |
| **SARS-Coronavirus** | | |  |  |  |  |  |  |  |  |  |  |  |
| There were no SNPs with 4 or more studies used for meta-analysis | | | | | | | | | | | | | |
| **Pneumonia** | |  |  |  |  |  |  |  |  |  |  |  |  |
| FCGR2A | | rs1801274 | AG | G | 6 | 1704 | 2211 | 1.05 [0.86-1.30] | 0.615 | 0.21 [0.00-0.65] | - | - | - |
| IL6 IL6R | | rs1800795 | CG | c | 6 | 3062 | 2778 | 0.96 [0.77-1.21] | 0.749 | 0.37 [0.00-0.75] | - | - | - |

Supplementary Table 5. Meta-analysis results, recessive model

| Gene | rs code | Heterozygote | Risk allele | N studies | N cases (allelels) | N Controls (allelels) | OR [95% CI] | P | I^2^ [95% CI] | Venice score | BFDP (med/low) | BFDP (very low) |
| --- | --- | --- | --- | --- | --- | --- | --- | --- | --- | --- | --- | --- |
| **Pooled result** |  |  |  |  |  |  |  |  |  |  |  |  |
| ACE | rs4340 | D/I | D | 6 | 1444 | 2113 | 1.02 [0.86-1.20] | 0.845 | 0.00 [0.00-0.32] | - | - | - |
| CCL2 | rs3760396 | CG | C | 4 | 1067 | 912 | 1.04 [0.75-1.44] | 0.823 | 0.00 [0.00-0.83] | - | - | - |
| CCL2 | rs3917891 | CT | T | 4 | 3056 | 3222 | 0.87 [0.48-1.57] | 0.635 | 0.35 [0.00-0.77] | - | - | - |
| CCL2 | rs2530797 | [CT] | C | 5 | 1508 | 1400 | 0.90 [0.74-1.10] | 0.316 | 0.00 [0.00-0.79] | - | - | - |
| CCL2 | rs1024610 | AT | T | 6 | 3475 | 3700 | 1.25 [0.92-1.70] | 0.149 | 0.00 [0.00-0.67] | - | - | - |
| CCL2 | rs2857656 | CG | C | 6 | 3266 | 3435 | 1.07 [0.89-1.29] | 0.463 | 0.50 [0.00-0.80] | - | - | - |
| CCL2 | rs4586 | CT | T | 8 | 3882 | 4338 | 0.97 [0.82-1.15] | 0.748 | 0.24 [0.00-0.66] | - | - | - |
| CCL2 | rs1024611 | AG | G | 18 | 7798 | 9078 | 1.18 [0.91-1.54] | 0.210 | 0.82 [0.73-0.88] | - | - | - |
| CCL5 | rs2280789 | CT | G | 8 | 2032 | 2473 | 1.11 [0.92-1.34] | 0.283 | 0.45 [0.00-0.76] | - | - | - |
| CCL5 | rs2280788 | CG | C | 8 | 2291 | 2673 | 2.33 [0.84-6.48] | 0.104 | 0.84 [0.70-0.91] | - | - | - |
| CCL5 | rs2107538 | [AG] | T | 14 | 3760 | 4653 | 0.98 [0.84-1.14] | 0.795 | 0.59 [0.27-0.77] | - | - | - |
| CCR2 | rs1799864 | AG | A | 4 | 978 | 1823 | 1.11 [0.85-1.45] | 0.447 | 0.44 [0.00-0.81] | - | - | - |
| CCR5 | rs1799987 | AG | A | 4 | 1307 | 2118 | 1.03 [0.87-1.22] | 0.754 | 0.00 [0.00-0.81] | - | - | - |
| CD14 | rs2569190 | CT | A | 14 | 2193 | 3662 | 1.23 [0.94-1.60] | 0.131 | 0.74 [0.56-0.85] | - | - | - |
| CD209 | rs735239 | AG | G | 4 | 1206 | 1320 | 0.93 [0.56-1.54] | 0.774 | 0.00 [0.00-0.26] | - | - | - |
| CD209 | rs735240 | AG | A | 5 | 1660 | 2555 | 1.13 [0.92-1.39] | 0.236 | 0.56 [0.00-0.84] | - | - | - |
| CD209 | rs2287886 | AG | A | 6 | 1658 | 2421 | 0.92 [0.72-1.18] | 0.512 | 0.54 [0.00-0.82] | - | - | - |
| CD209 | rs4804803 | AG | G | 9 | 2323 | 3134 | 1.04 [0.76-1.44] | 0.791 | 0.14 [0.00-0.56] | - | - | - |
| FCGR2A | rs1801274 | AG | G | 8 | 1915 | 2459 | 1.27 [0.83-1.94] | 0.273 | 0.81 [0.64-0.90] | - | - | - |
| IFNG | rs1861494 | AG | C | 5 | 2168 | 2528 | 0.62 [0.34-1.12] | 0.115 | 0.79 [0.49-0.91] | - | - | - |
| IFNG | rs2430561 | AT | A | 24 | 4285 | 5072 | 0.82 [0.68-0.98] | 0.033 | 0.42 [0.05-0.64] | BBC | 0.865 | 0.997 |
| IL10 | rs1800871 | CT | A | 14 | 3645 | 5249 | 0.87 [0.73-1.05] | 0.142 | 0.41 [0.00-0.69] | - | - | - |
| IL10 | rs1800872 | AC | T | 19 | 4170 | 7099 | 1.07 [0.91-1.26] | 0.437 | 0.61 [0.36-0.76] | - | - | - |
| IL10 | rs1800896 | AG | C | 26 | 5662 | 8596 | 1.26 [1.03-1.56] | 0.027 | 0.51 [0.23-0.69] | BCC | 0.875 | 0.997 |
| IL12B | rs3212227 | AC | G | 9 | 2535 | 3371 | 1.01 [0.80-1.29] | 0.908 | 0.36 [0.00-0.71] | - | - | - |
| IL12RB1 | rs375947 | [CT] | G | 4 | 1022 | 2025 | 0.89 [0.70-1.14] | 0.353 | 0.40 [0.00-0.80] | - | - | - |
| IL18 | rs187238 | CG | G | 5 | 1087 | 1774 | 1.02 [0.84-1.24] | 0.834 | 0.00 [0.00-0.00] | - | - | - |
| IL18 | rs1946518 | [AC] | T | 7 | 1768 | 3007 | 0.90 [0.72-1.11] | 0.304 | 0.51 [0.00-0.79] | - | - | - |
| IL1B | rs1143634 | [CT] | A | 7 | 1361 | 2016 | 1.35 [0.74-2.48] | 0.325 | 0.74 [0.45-0.88] | - | - | - |
| IL1B | rs16944 | [CT] | A | 8 | 1966 | 2909 | 1.23 [1.03-1.47] | 0.023 | 0.23 [0.00-0.65] | BAC | 0.843 | 0.996 |
| IL2 | rs2069762 | GT | C | 6 | 973 | 2213 | 0.91 [0.72-1.15] | 0.436 | 0.46 [0.00-0.79] | - | - | - |
| IL4 | rs2070874 | CT | T | 4 | 1059 | 2210 | 1.66 [1.29-2.14] | 8.68E-05 | 0.00 [0.00-0.39] | BAC | 0.128 | 0.886 |
| IL4 | rs2243248 | GT | G | 4 | 653 | 1205 | 1.50 [0.66-3.39] | 0.334 | 0.90 [0.77-0.96] | - | - | - |
| IL4 | rs2243250 | CT | T | 11 | 2030 | 3870 | 1.15 [0.92-1.44] | 0.216 | 0.33 [0.00-0.67] | - | - | - |
| IL4RA | rs1801275 | [AG] | G | 5 | 908 | 2160 | 0.80 [0.55-1.18] | 0.266 | 0.00 [0.00-0.71] | - | - | - |
| IL6 IL6R | rs1800797 | AG | A | 4 | 1017 | 1789 | 1.17 [0.95-1.44] | 0.131 | 0.00 [0.00-0.80] | - | - | - |
| IL6 IL6R | rs1800795 | CG | C | 12 | 4246 | 4776 | 1.20 [1.03-1.41] | 0.024 | 0.48 [0.00-0.73] | BBC | 0.861 | 0.997 |
| IL8 | rs4073 | AT | T | 8 | 1769 | 2943 | 1.03 [0.80-1.31] | 0.842 | 0.60 [0.13-0.82] | - | - | - |
| MBL2 | rs7095891 | CT | A | 6 | 2157 | 4175 | 1.03 [0.89-1.19] | 0.689 | 0.00 [0.00-0.00] | - | - | - |
| MBL2 | rs11003125 | CG | C | 8 | 2379 | 4449 | 1.00 [0.81-1.23] | 0.998 | 0.28 [0.00-0.68] | - | - | - |
| MBL2 | rs1800450 | AG (AB) | T | 9 | 1230 | 2231 | 0.93 [0.66-1.31] | 0.670 | 0.63 [0.23-0.82] | - | - | - |
| MBL2 | rs7096206 | CG | G | 9 | 2590 | 4799 | 1.00 [0.81-1.24] | 0.994 | 0.55 [0.05-0.79] | - | - | - |
| MIF | rs755622 | CG | C | 4 | 2207 | 1273 | 0.76 [0.53-1.10] | 0.150 | 0.79 [0.43-0.92] | - | - | - |
| OAS1 | rs2660 | AG | A | 5 | 7493 | 10512 | 0.98 [0.90-1.07] | 0.666 | 0.04 [0.00-0.80] | - | - | - |
| P2RX7 | rs2393799 | CT | T | 11 | 2465 | 3047 | 1.17 [0.87-1.58] | 0.290 | 0.70 [0.45-0.84] | - | - | - |
| P2RX7 | rs3751143 | AC | C | 13 | 2263 | 2961 | 1.06 [0.77-1.45] | 0.737 | 0.00 [0.00-0.56] | - | - | - |
| SFTPC | rs1124 | [AG] | A | 4 | 692 | 925 | 0.89 [0.62-1.27] | 0.518 | 0.32 [0.00-0.76] | - | - | - |
| SLC11A1 | rs17235409 | AG | A | 26 | 3891 | 4278 | 0.83 [0.69-1.00] | 0.047 | 0.61 [0.40-0.74] | BCC | 0.903 | 0.998 |
| SLC11A1 | rs17235416 | +/del | - | 20 | 4357 | 4965 | 1.27 [0.96-1.68] | 0.097 | 0.36 [0.00-0.63] | - | - | - |
| SLC11A1 | rs3731865 | CG | C | 16 | 2472 | 2787 | 0.70 [0.54-0.92] | 0.010 | 0.69 [0.49-0.82] | BCC | 0.782 | 0.995 |
| SP-A1 | rs1059047 | CT | C | 4 | 1141 | 1766 | 0.94 [0.66-1.36] | 0.757 | 0.56 [0.00-0.85] | - | - | - |
| SP-A1 | rs1136450 | CG | G | 4 | 1141 | 1766 | 0.78 [0.65-0.92] | 0.005 | 0.00 [0.00-0.81] | BAC | 0.547 | 0.984 |
| SP-A1 | rs4253527 | CT | T | 4 | 1141 | 1766 | 0.54 [0.29-1.00] | 0.051 | 0.00 [0.00-0.70] | - | - | - |
| SP-A2 | rs1059046 | AC | A | 4 | 1141 | 1766 | 1.36 [1.08-1.72] | 0.010 | 0.00 [0.00-0.68] | BAC | 0.763 | 0.994 |
| SP-A2 | rs17886395 | CG | G | 4 | 1141 | 1766 | 0.95 [0.62-1.45] | 0.799 | 0.75 [0.30-0.91] | - | - | - |
| SP-A2 | rs1965708 | AC | T | 4 | 1141 | 1766 | 0.85 [0.71-1.01] | 0.059 | 0.00 [0.00-0.29] | - | - | - |
| SP-D | rs721917 | CT | G | 5 | 1793 | 3148 | 1.01 [0.77-1.31] | 0.950 | 0.71 [0.27-0.89] | - | - | - |
| TIRAP | rs8177374 | CT | T | 6 | 6483 | 7643 | 1.03 [0.70-1.52] | 0.871 | 0.24 [0.00-0.67] | - | - | - |
| TLR1 | rs5743618 | GT | C | 5 | 1011 | 1373 | 1.07 [0.67-1.72] | 0.782 | 0.00 [0.00-0.77] | - | - | - |
| TLR2 | rs3804099 | CT | C | 7 | 1602 | 1979 | 0.90 [0.73-1.10] | 0.288 | 0.40 [0.00-0.75] | - | - | - |
| TLR2 | rs5743708 | AG | A | 10 | 2688 | 3793 | 0.85 [0.49-1.47] | 0.554 | 0.64 [0.29-0.82] | - | - | - |
| TLR4 | rs4986791 | [CT] | T | 10 | 2599 | 5499 | 1.18 [0.79-1.76] | 0.423 | 0.00 [0.00-0.37] | - | - | - |
| TLR4 | rs4986790 | AG | G | 11 | 3504 | 11917 | 1.22 [0.82-1.82] | 0.335 | 0.00 [0.00-0.45] | - | - | - |
| TLR6 | rs5743810 | CT | A | 5 | 978 | 2058 | 1.32 [1.08-1.62] | 0.007 | 0.00 [0.00-0.59] | CAC | 0.717 | 0.993 |
| TLR9 | rs187084 | [CT] | G | 4 | 499 | 688 | 0.94 [0.74-1.19] | 0.590 | 0.00 [0.00-0.69] | - | - | - |
| TNFA | rs1800630 | AC | A | 5 | 993 | 1255 | 1.05 [0.88-1.27] | 0.579 | 0.00 [0.00-0.73] | - | - | - |
| TNFA | rs1799724 | CT | T | 7 | 1465 | 1979 | 0.47 [0.20-1.08] | 0.074 | 0.45 [0.00-0.77] | - | - | - |
| TNFA | 488 | AG | A | 7 | 3960 | 3901 | 0.99 [0.74-1.33] | 0.955 | 0.57 [0.00-0.81] | - | - | - |
| TNFA | rs361525 | AG | A | 18 | 3439 | 5198 | 0.65 [0.44-0.96] | 0.032 | 0.83 [0.74-0.89] | BCC | 0.890 | 0.998 |
| TNFA | rs1800629 | AG | A | 28 | 4858 | 6984 | 0.92 [0.80-1.06] | 0.271 | 0.35 [0.00-0.59] | - | - | - |
| TNFB_LTA | rs909253 | [AG] | G | 4 | 1468 | 1568 | 0.94 [0.54-1.64] | 0.838 | 0.43 [0.00-0.81] | - | - | - |
| VDR | rs7975232 | Aa | A | 9 | 1005 | 1495 | 0.99 [0.74-1.34] | 0.960 | 0.14 [0.00-0.56] | - | - | - |
| VDR | rs1544410 | Bb | T | 13 | 1983 | 2802 | 0.96 [0.74-1.25] | 0.753 | 0.61 [0.28-0.78] | - | - | - |
| VDR | rs731236 | Tt | G | 21 | 3296 | 4640 | 1.08 [0.82-1.43] | 0.575 | 0.42 [0.03-0.66] | - | - | - |
| VDR | rs2228570 | Ff | A | 21 | 2327 | 2785 | 1.42 [1.05-1.93] | 0.024 | 0.47 [0.12-0.68] | BBC | 0.865 | 0.997 |
| **Tuberculosis** |  |  |  |  |  |  |  |  |  |  |  |  |
| CCL2 | rs3917891 | CT | T | 4 | 3056 | 3222 | 0.87 [0.48-1.57] | 0.635 | 0.35 [0.00-0.77] | - | - | - |
| CCL2 | rs4586 | CT | T | 8 | 3882 | 4338 | 0.97 [0.82-1.15] | 0.748 | 0.24 [0.00-0.66] | - | - | - |
| CCL2 | rs1024611 | AG | G | 17 | 7391 | 8070 | 1.18 [0.89-1.57] | 0.241 | 0.83 [0.75-0.89] | - | - | - |
| CCL5 | rs2280788 | CG | C | 5 | 1275 | 1407 | 3.56 [0.54-23.48] | 0.188 | 0.89 [0.77-0.95] | - | - | - |
| CCL5 | rs2107538 | AG | T | 8 | 2017 | 2107 | 0.96 [0.76-1.22] | 0.746 | 0.68 [0.33-0.85] | - | - | - |
| CD14 | rs2569190 | CT | A | 6 | 1072 | 1327 | 1.41 [0.86-2.30] | 0.174 | 0.84 [0.67-0.92] | - | - | - |
| CD209 | rs2287886 | AG | A | 4 | 1082 | 1243 | 0.87 [0.59-1.30] | 0.501 | 0.70 [0.13-0.89] | - | - | - |
| CD209 | rs735239 | AG | G | 4 | 1206 | 1320 | 0.93 [0.56-1.54] | 0.774 | 0.00 [0.00-0.26] | - | - | - |
| CD209 | rs4804803 | AG | G | 8 | 1735 | 1956 | 1.12 [0.77-1.62] | 0.564 | 0.16 [0.00-0.59] | - | - | - |
| IFNG | rs1861494 | AG | C | 5 | 2168 | 2528 | 0.62 [0.34-1.12] | 0.115 | 0.79 [0.49-0.91] | - | - | - |
| IFNG | rs2430561 | AT | A | 23 | 4188 | 4655 | 0.81 [0.66-0.98] | 0.033 | 0.44 [0.08-0.66] | BBC | 0.867 | 0.997 |
| IL10 | rs1800871 | CT | A | 13 | 3580 | 5203 | 0.90 [0.76-1.07] | 0.236 | 0.35 [0.00-0.67] | - | - | - |
| IL10 | rs1800872 | AC | T | 14 | 3322 | 5170 | 1.03 [0.85-1.25] | 0.774 | 0.62 [0.32-0.79] | - | - | - |
| IL10 | rs1800896 | AG | C | 22 | 4893 | 6811 | 1.37 [1.04-1.80] | 0.024 | 0.56 [0.29-0.73] | BCC | 0.854 | 0.997 |
| IL12B | rs3212227 | AC | G | 8 | 2128 | 2363 | 0.98 [0.75-1.26] | 0.852 | 0.38 [0.00-0.73] | - | - | - |
| IL18 | rs1946518 | AC | T | 5 | 1267 | 1905 | 0.88 [0.66-1.18] | 0.387 | 0.63 [0.02-0.86] | - | - | - |
| IL1B | rs1143634 | [CT] | A | 6 | 954 | 1008 | 1.60 [0.81-3.14] | 0.173 | 0.70 [0.28-0.87] | - | - | - |
| IL1B | rs16944 | [CT] | A | 6 | 1509 | 1725 | 1.26 [0.98-1.62] | 0.071 | 0.44 [0.00-0.78] | - | - | - |
| IL2 | rs2069762 | GT | C | 5 | 566 | 1205 | 0.84 [0.63-1.12] | 0.242 | 0.39 [0.00-0.77] | - | - | - |
| IL4 | rs2243250 | CT | T | 7 | 1134 | 1816 | 1.07 [0.78-1.48] | 0.661 | 0.44 [0.00-0.77] | - | - | - |
| IL6 IL6R | rs1800795 | CG | C | 4 | 712 | 944 | 1.50 [0.84-2.67] | 0.171 | 0.60 [0.00-0.87] | - | - | - |
| MBL2 | rs1800450 | AG (AB) | T | 5 | 622 | 951 | 1.15 [0.84-1.57] | 0.398 | 0.16 [0.00-0.83] | - | - | - |
| MBL2 | rs7096206 | CG (XY) | G | 5 | 1589 | 3079 | 0.95 [0.66-1.36] | 0.786 | 0.67 [0.14-0.87] | - | - | - |
| P2RX7 | rs2393799 | CT | T | 11 | 2465 | 3047 | 1.17 [0.87-1.58] | 0.290 | 0.70 [0.45-0.84] | - | - | - |
| P2RX7 | rs3751143 | AC | C | 13 | 2263 | 2961 | 1.06 [0.77-1.45] | 0.737 | 0.00 [0.00-0.56] | - | - | - |
| SLC11A1 | rs17235409 | AG | A | 26 | 3891 | 4278 | 0.83 [0.69-1.00] | 0.047 | 0.61 [0.40-0.74] | BCC | 0.903 | 0.998 |
| SLC11A1 | rs17235416 | +/del | - | 20 | 4357 | 4965 | 1.27 [0.96-1.68] | 0.097 | 0.36 [0.00-0.63] | - | - | - |
| SLC11A1 | rs3731865 | CG | C | 16 | 2472 | 2787 | 0.70 [0.54-0.92] | 0.010 | 0.69 [0.49-0.82] | BCC | 0.782 | 0.995 |
| TIRAP | rs8177374 | CT | T | 6 | 6483 | 7643 | 1.03 [0.70-1.52] | 0.871 | 0.24 [0.00-0.67] | - | - | - |
| TLR2 | rs5743708 | AG | A | 4 | 1631 | 1562 | 0.30 [0.18-0.50] | 4.77E-06 | 0.00 [0.00-0.83] | BAC | 0.282 | 0.954 |
| TLR2 | rs3804099 | CT | C | 5 | 1361 | 1449 | 0.87 [0.66-1.15] | 0.327 | 0.60 [0.00-0.85] | - | - | - |
| TLR4 | rs4986791 | CT | T | 4 | 1133 | 1104 | 1.29 [0.70-2.36] | 0.410 | 0.12 [0.00-0.87] | - | - | - |
| TLR4 | rs4986790 | AG | G | 4 | 1714 | 2008 | 1.41 [0.64-3.12] | 0.390 | 0.38 [0.00-0.79] | - | - | - |
| TNFA | rs1800630 | AC | A | 4 | 918 | 1124 | 1.05 [0.86-1.27] | 0.637 | 0.00 [0.00-0.85] | - | - | - |
| TNFA | rs1799724 | CT | T | 5 | 983 | 840 | 0.33 [0.13-0.83] | 0.019 | 0.36 [0.00-0.76] | CBC | 0.930 | 0.999 |
| TNFA | rs361525 | AG | A | 11 | 1398 | 2239 | 0.62 [0.32-1.20] | 0.159 | 0.88 [0.80-0.92] | - | - | - |
| TNFA | rs1800629 | AG | A | 19 | 2527 | 3722 | 0.90 [0.75-1.09] | 0.301 | 0.38 [0.00-0.64] | - | - | - |
| VDR | rs7975232 | Aa | A | 8 | 909 | 1394 | 0.98 [0.72-1.32] | 0.878 | 0.14 [0.00-0.57] | - | - | - |
| VDR | rs1544410 | Bb | T | 12 | 1576 | 1794 | 0.96 [0.71-1.30] | 0.792 | 0.63 [0.31-0.80] | - | - | - |
| VDR | rs731236 | Tt | G | 18 | 2737 | 3465 | 1.08 [0.76-1.53] | 0.666 | 0.46 [0.06-0.69] | - | - | - |
| VDR | rs2228570 | Ff | A | 19 | 2170 | 2620 | 1.38 [1.02-1.86] | 0.035 | 0.43 [0.02-0.67] | BBC | 0.883 | 0.997 |
| **Influenza** |  |  |  |  |  |  |  |  |  |  |  |  |
| There were no SNPs with 4 or more studies used for meta-analysis | | | | | | | | | | | | |
| **Respiratory syncytial virus** | |  |  |  |  |  |  |  |  |  |  |  |
| CCL5 | rs2107538 | [AG] | T | 4 | 892 | 1601 | 1.02 [0.71-1.46] | 0.929 | 0.70 [0.15-0.90] | - | - | - |
| CD14 | rs2569190 | CT | A | 5 | 814 | 1658 | 1.24 [0.83-1.83] | 0.291 | 0.61 [0.00-0.85] | - | - | - |
| IL4 | rs2243250 | CT | T | 4 | 896 | 2054 | 1.31 [0.98-1.76] | 0.069 | 0.00 [0.00-0.83] | - | - | - |
| IL8 | rs4073 | AT | T | 4 | 1193 | 1937 | 1.07 [0.71-1.62] | 0.736 | 0.81 [0.50-0.93] | - | - | - |
| TLR4 | rs4986791 | [CT] | T | 4 | 1039 | 3752 | 0.96 [0.41-2.27] | 0.935 | 0.00 [0.00-0.73] | - | - | - |
| TLR4 | rs4986790 | [AG] | G | 6 | 1363 | 9266 | 0.83 [0.31-2.22] | 0.709 | 0.00 [0.00-0.32] | - | - | - |
| **SARS-Coronavirus** | |  |  |  |  |  |  |  |  |  |  |  |
| There were no SNPs with 4 or more studies used for meta-analysis | | | | | | | | | | | | |
| **Pneumonia** |  |  |  |  |  |  |  |  |  |  |  |  |
| FCGR2A | rs1801274 | AG | G | 6 | 1704 | 2211 | 1.65 [1.05-2.59] | 0.031 | 0.79 [0.55-0.91] | BCC | 0.899 | 0.998 |
| IL6 IL6R | rs1800795 | CG | C | 6 | 3062 | 2778 | 1.08 [0.94-1.25] | 0.288 | 0.29 [0.00-0.71] | - | - | - |

Supplementary Table 6. Meta-analysis results, heterozygote advantage model

| Gene | rs code | Heterozygote | | Risk allele | | N studies | | | N cases (allelels) | | N Controls (allelels) | | | OR [95% CI] | | P | I^2^ [95% CI] | | | Venice score | | BFDP (med/low) | BFDP (very low) |
| --- | --- | --- | --- | --- | --- | --- | --- | --- | --- | --- | --- | --- | --- | --- | --- | --- | --- | --- | --- | --- | --- | --- | --- |
| **Pooled result** |  |  | |  | |  | | |  | |  | | |  | |  |  | | |  | |  |  |
| ACE | rs4340 | D/I | | D | | 6 | | | 1444 | | 2113 | | | 0.89 [0.76-1.05] | | 0.167 | 0.25 [0.00-0.68] | | | - | | - | - |
| CCL2 | rs3760396 | CG | | C | | 4 | | | 1067 | | 912 | | | 1.01 [0.72-1.40] | | 0.960 | 0.00 [0.00-0.73] | | | - | | - | - |
| CCL2 | rs3917891 | CT | | T | | 5 | | | 3056 | | 3222 | | | 0.85 [0.76-0.95] | | 0.004 | 0.00 [0.00-0.79] | | | BAC | | 0.613 | 0.988 |
| CCL2 | rs2530797 | [CT] | | C | | 5 | | | 1508 | | 1400 | | | 1.06 [0.88-1.29] | | 0.524 | 0.18 [0.00-0.83] | | | - | | - | - |
| CCL2 | rs1024610 | AT | | T | | 6 | | | 3475 | | 3700 | | | 0.94 [0.81-1.10] | | 0.464 | 0.00 [0.00-0.74] | | | - | | - | - |
| CCL2 | rs2857656 | CG | | C | | 6 | | | 3266 | | 3435 | | | 0.93 [0.85-1.03] | | 0.150 | 0.00 [0.00-0.64] | | | - | | - | - |
| CCL2 | rs4586 | CT | | T | | 8 | | | 3882 | | 4338 | | | 1.11 [1.02-1.22] | | 0.019 | 0.00 [0.00-0.43] | | | BAC | | 0.901 | 0.998 |
| CCL2 | rs1024611 | AG | | G | | 18 | | | 7798 | | 9078 | | | 0.95 [0.86-1.05] | | 0.321 | 0.50 [0.14-0.71] | | | - | | - | - |
| CCL5 | rs2280789 | CT | | G | | 8 | | | 2032 | | 2473 | | | 0.90 [0.74-1.09] | | 0.291 | 0.46 [0.00-0.76] | | | - | | - | - |
| CCL5 | rs2280788 | CG | | C | | 10 | | | 2291 | | 2673 | | | 1.12 [0.69-1.82] | | 0.646 | 0.81 [0.66-0.89] | | | - | | - | - |
| CCL5 | rs2107538 | [AG] | | T | | 14 | | | 3760 | | 4653 | | | 0.91 [0.77-1.09] | | 0.322 | 0.69 [0.46-0.82] | | | - | | - | - |
| CCR2 | rs1799864 | AG | | A | | 4 | | | 978 | | 1823 | | | 0.89 [0.71-1.10] | | 0.277 | 0.10 [0.00-0.86] | | | - | | - | - |
| CCR5 | rs1799987 | AG | | A | | 4 | | | 1307 | | 2118 | | | 0.99 [0.86-1.14] | | 0.900 | 0.00 [0.00-0.69] | | | - | | - | - |
| CD14 | rs2569190 | CT | | A | | 14 | | | 2193 | | 3662 | | | 0.92 [0.83-1.03] | | 0.163 | 0.00 [0.00-0.53] | | | - | | - | - |
| CD209 | rs735239 | AG | | G | | 4 | | | 1206 | | 1320 | | | 0.62 [0.44-0.87] | | 0.006 | 0.67 [0.03-0.89] | | | BCC | | 0.757 | 0.994 |
| CD209 | rs735240 | AG | | A | | 5 | | | 1660 | | 2555 | | | 0.95 [0.72-1.24] | | 0.684 | 0.74 [0.35-0.89] | | | - | | - | - |
| CD209 | rs2287886 | AG | | A | | 6 | | | 1658 | | 2421 | | | 0.97 [0.84-1.12] | | 0.659 | 0.14 [0.00-0.78] | | | - | | - | - |
| CD209 | rs4804803 | AG | | G | | 10 | | | 2323 | | 3134 | | | 1.01 [0.82-1.23] | | 0.937 | 0.57 [0.13-0.79] | | | - | | - | - |
| FCGR2A | rs1801274 | AG | | G | | 8 | | | 1915 | | 2459 | | | 0.80 [0.65-1.00] | | 0.049 | 0.46 [0.00-0.76] | | | BBC | | 0.900 | 0.998 |
| IFNG | rs1861494 | AG | | C | | 5 | | | 2168 | | 2528 | | | 1.21 [0.98-1.49] | | 0.077 | 0.50 [0.00-0.82] | | | - | | - | - |
| IFNG | rs2430561 | AT | | A | | 24 | | | 4285 | | 5072 | | | 0.88 [0.78-1.00] | | 0.047 | 0.39 [0.00-0.63] | | | BBC | | 0.917 | 0.998 |
| IL10 | rs1800871 | CT | | A | | 14 | | | 3645 | | 5249 | | | 1.18 [0.96-1.45] | | 0.119 | 0.73 [0.53-0.84] | | | - | | - | - |
| IL10 | rs1800872 | AC | | T | | 19 | | | 4170 | | 7099 | | | 0.88 [0.76-1.01] | | 0.073 | 0.57 [0.29-0.74] | | | - | | - | - |
| IL10 | rs1800896 | AG | | C | | 27 | | | 5662 | | 8596 | | | 0.84 [0.73-0.96] | | 0.009 | 0.56 [0.33-0.72] | | | BCC | | 0.760 | 0.994 |
| IL12B | rs3212227 | AC | | G | | 9 | | | 2535 | | 3371 | | | 1.02 [0.91-1.15] | | 0.696 | 0.11 [0.00-0.69] | | | - | | - | - |
| IL12RB1 | rs375947 | [CT] | | G | | 4 | | | 1022 | | 2025 | | | 1.08 [0.93-1.26] | | 0.324 | 0.00 [0.00-0.00] | | | - | | - | - |
| IL18 | rs187238 | CG | | G | | 5 | | | 1087 | | 1774 | | | 0.97 [0.82-1.16] | | 0.763 | 0.00 [0.00-0.01] | | | - | | - | - |
| IL18 | rs1946518 | [AC] | | T | | 7 | | | 1768 | | 3007 | | | 0.97 [0.86-1.09] | | 0.602 | 0.00 [0.00-0.00] | | | - | | - | - |
| IL1A | rs1800587 | CT | | A | | 4 | | | 615 | | 1516 | | | 1.10 [0.89-1.36] | | 0.368 | 0.02 [0.00-0.85] | | | - | | - | - |
| IL1B | rs1143634 | [CT] | | A | | 7 | | | 1361 | | 2016 | | | 1.01 [0.73-1.39] | | 0.951 | 0.62 [0.12-0.83] | | | - | | - | - |
| IL1B | rs16944 | [CT] | | A | | 8 | | | 1966 | | 2909 | | | 0.96 [0.84-1.09] | | 0.504 | 0.06 [0.00-0.70] | | | - | | - | - |
| IL2 | rs2069762 | GT | | C | | 6 | | | 973 | | 2213 | | | 1.07 [0.80-1.43] | | 0.660 | 0.64 [0.14-0.85] | | | - | | - | - |
| IL4 | rs2243248 | GT | | G | | 4 | | | 653 | | 1205 | | | 0.66 [0.31-1.41] | | 0.283 | 0.88 [0.73-0.95] | | | - | | - | - |
| IL4 | rs2070874 | CT | | T | | 5 | | | 1059 | | 2210 | | | 0.88 [0.68-1.14] | | 0.344 | 0.50 [0.00-0.82] | | | - | | - | - |
| IL4 | rs2243250 | CT | | T | | 11 | | | 2030 | | 3870 | | | 0.98 [0.77-1.26] | | 0.898 | 0.75 [0.54-0.86] | | | - | | - | - |
| IL4RA | rs1801275 | [AG] | | G | | 5 | | | 908 | | 2160 | | | 1.00 [0.84-1.18] | | 0.971 | 0.00 [0.00-0.72] | | | - | | - | - |
| IL6 IL6R | rs1800797 | AG | | A | | 4 | | | 1017 | | 1789 | | | 0.85 [0.69-1.04] | | 0.105 | 0.00 [0.00-0.60] | | | - | | - | - |
| IL6 IL6R | rs1800795 | CG | | C | | 12 | | | 4246 | | 4776 | | | 0.85 [0.73-0.99] | | 0.039 | 0.44 [0.00-0.71] | | | BBC | | 0.889 | 0.998 |
| IL8 | rs4073 | AT | | T | | 8 | | | 1769 | | 2943 | | | 1.03 [0.91-1.16] | | 0.686 | 0.00 [0.00-0.68] | | | - | | - | - |
| MBL2 | rs7095891 | CT | | A | | 6 | | | 2157 | | 4175 | | | 0.89 [0.80-1.00] | | 0.044 | 0.00 [0.00-0.74] | | | BAC | | 0.921 | 0.998 |
| MBL2 | rs11003125 | CG | | C | | 8 | | | 2379 | | 4449 | | | 0.95 [0.84-1.06] | | 0.363 | 0.00 [0.00-0.59] | | | - | | - | - |
| MBL2 | rs1800450 | AG (AB) | | T | | 9 | | | 1230 | | 2231 | | | 1.12 [0.82-1.54] | | 0.465 | 0.61 [0.20-0.81] | | | - | | - | - |
| MBL2 | rs7096206 | CG | | G | | 9 | | | 2590 | | 4799 | | | 0.93 [0.78-1.10] | | 0.398 | 0.49 [0.00-0.76] | | | - | | - | - |
| MIF | rs755622 | CG | | C | | 4 | | | 2207 | | 1273 | | | 1.10 [0.85-1.42] | | 0.468 | 0.54 [0.00-0.85] | | | - | | - | - |
| OAS1 | rs2660 | AG | | A | | 5 | | | 7493 | | 10512 | | | 1.00 [0.91-1.08] | | 0.922 | 0.23 [0.00-0.68] | | | - | | - | - |
| P2RX7 | rs2393799 | CT | | T | | 11 | | | 2465 | | 3047 | | | 1.03 [0.89-1.18] | | 0.726 | 0.22 [0.00-0.61] | | | - | | - | - |
| P2RX7 | rs3751143 | AC | | C | | 13 | | | 2263 | | 2961 | | | 1.30 [1.06-1.59] | | 0.011 | 0.56 [0.17-0.76] | | | BCC | | 0.758 | 0.994 |
| PTPN22 | rs2476601 | CT | | A | | 4 | | | 604 | | 1373 | | | 0.76 [0.35-1.64] | | 0.485 | 0.64 [0.00-0.88] | | | - | | - | - |
| SFTPC | rs1124 | [AG] | | A | | 4 | | | 692 | | 925 | | | 1.19 [0.91-1.55] | | 0.214 | 0.16 [0.00-0.87] | | | - | | - | - |
| SLC11A1 | rs17235409 | AG | | A | | 26 | | | 3891 | | 4278 | | | 1.15 [0.95-1.39] | | 0.150 | 0.60 [0.39-0.74] | | | - | | - | - |
| SLC11A1 | rs17235416 | +/del | | - | | 23 | | | 4357 | | 4965 | | | 1.20 [1.04-1.39] | | 0.014 | 0.47 [0.13-0.67] | | | BBC | | 0.804 | 0.995 |
| SLC11A1 | rs3731865 | CG | | C | | 16 | | | 2472 | | 2787 | | | 1.24 [1.00-1.54] | | 0.051 | 0.52 [0.15-0.73] | | | - | | - | - |
| SP-A1 | rs1059047 | CT | | C | | 4 | | | 1141 | | 1766 | | | 1.04 [0.74-1.45] | | 0.840 | 0.48 [0.00-0.83] | | | - | | - | - |
| SP-A1 | rs1136450 | CG | | G | | 4 | | | 1141 | | 1766 | | | 1.19 [1.01-1.40] | | 0.042 | 0.00 [0.00-0.31] | | | BAC | | 0.885 | 0.998 |
| SP-A1 | rs4253527 | CT | | T | | 4 | | | 1141 | | 1766 | | | 1.18 [0.90-1.55] | | 0.241 | 0.32 [0.00-0.76] | | | - | | - | - |
| SP-A2 | rs1059046 | AC | | A | | 4 | | | 1141 | | 1766 | | | 1.07 [0.90-1.25] | | 0.448 | 0.00 [0.00-0.52] | | | - | | - | - |
| SP-A2 | rs17886395 | CG | | G | | 4 | | | 1141 | | 1766 | | | 1.31 [1.06-1.61] | | 0.012 | 0.09 [0.00-0.86] | | | BAC | | 0.754 | 0.994 |
| SP-A2 | rs1965708 | AC | | T | | 4 | | | 1141 | | 1766 | | | 1.15 [0.96-1.38] | | 0.119 | 0.00 [0.00-0.00] | | | - | | - | - |
| SP-D | rs721917 | CT | | G | | 5 | | | 1793 | | 3148 | | | 0.92 [0.74-1.15] | | 0.473 | 0.61 [0.00-0.85] | | | - | | - | - |
| TIRAP | rs8177374 | CT | | T | | 10 | | | 6483 | | 7643 | | | 0.82 [0.62-1.10] | | 0.184 | 0.69 [0.39-0.84] | | | - | | - | - |
| TLR1 | rs5743618 | GT | | C | | 5 | | | 1011 | | 1373 | | | 0.74 [0.48-1.15] | | 0.185 | 0.62 [0.00-0.86] | | | - | | - | - |
| TLR2 | rs3804099 | CT | | C | | 7 | | | 1602 | | 1979 | | | 0.92 [0.75-1.12] | | 0.417 | 0.48 [0.00-0.78] | | | - | | - | - |
| TLR2 | rs5743708 | AG | | A | | 11 | | | 2688 | | 3793 | | | 1.05 [0.63-1.75] | | 0.842 | 0.57 [0.15-0.78] | | | - | | - | - |
| TLR4 | rs4986791 | [CT] | | T | | 12 | | | 2599 | | 5499 | | | 1.48 [0.63-3.44] | | 0.365 | 0.95 [0.93-0.96] | | | - | | - | - |
| TLR4 | rs4986790 | AG | | G | | 17 | | | 3504 | | 11917 | | | 1.23 [0.71-2.12] | | 0.455 | 0.92 [0.88-0.94] | | | - | | - | - |
| TLR5 | rs5744168 | CT | | A | | 4 | | | 862 | | 2163 | | | 1.08 [0.65-1.79] | | 0.776 | 0.68 [0.08-0.89] | | | - | | - | - |
| TLR6 | rs5743810 | CT | | A | | 5 | | | 978 | | 2058 | | | 0.89 [0.75-1.05] | | 0.172 | 0.00 [0.00-0.00] | | | - | | - | - |
| TLR9 | rs187084 | [CT] | | G | | 4 | | | 499 | | 688 | | | 1.12 [0.89-1.42] | | 0.342 | 0.00 [0.00-0.00] | | | - | | - | - |
| TNFA | rs1800630 | AC | | A | | 5 | | | 993 | | 1255 | | | 0.85 [0.71-1.03] | | 0.101 | 0.00 [0.00-0.39] | | | - | | - | - |
| TNFA | rs1799724 | CT | | T | | 7 | | | 1465 | | 1979 | | | 1.15 [0.77-1.71] | | 0.495 | 0.78 [0.54-0.89] | | | - | | - | - |
| TNFA | rs361525 | AG | | A | | 19 | | | 3439 | | 5198 | | | 1.28 [0.96-1.72] | | 0.098 | 0.66 [0.44-0.79] | | | - | | - | - |
| TNFA | rs1800629 | AG | | A | | 35 | | | 8818 | | 10885 | | | 1.05 [0.92-1.20] | | 0.498 | 0.29 [0.00-0.55] | | | - | | - | - |
| TNFB_LTA | rs909253 | [AG] | | G | | 4 | | | 1468 | | 1568 | | | 1.69 [0.93-3.04] | | 0.083 | 0.85 [0.63-0.94] | | | - | | - | - |
| VDR | rs7975232 | Aa | | A | | 9 | | | 1005 | | 1495 | | | 1.03 [0.80-1.33] | | 0.820 | 0.49 [0.00-0.76] | | | - | | - | - |
| VDR | rs1544410 | Bb | | T | | 13 | | | 1983 | | 2802 | | | 1.11 [0.94-1.33] | | 0.225 | 0.35 [0.00-0.66] | | | - | | - | - |
| VDR | rs731236 | Tt | | G | | 21 | | | 3296 | | 4640 | | | 1.11 [0.96-1.28] | | 0.144 | 0.31 [0.00-0.60] | | | - | | - | - |
| VDR | rs2228570 | Ff | | A | | 21 | | | 2327 | | 2785 | | | 0.88 [0.72-1.07] | | 0.195 | 0.59 [0.33-0.74] | | | - | | - | - |
| **Tuberculosis** |  |  | |  | |  | | |  | |  | | |  | |  |  | | |  | |  |  |
| CCL2 | rs3917891 | CT | | T | | 5 | | | 3056 | | 3222 | | | 0.85 [0.76-0.95] | | 0.004 | 0.00 [0.00-0.79] | | | BAC | | 0.613 | 0.988 |
| CCL2 | rs4586 | CT | | T | | 8 | | | 3882 | | 4338 | | | 1.11 [1.02-1.22] | | 0.019 | 0.00 [0.00-0.43] | | | BAC | | 0.901 | 0.998 |
| CCL2 | rs1024611 | AG | | G | | 17 | | | 7391 | | 8070 | | | 0.96 [0.86-1.07] | | 0.420 | 0.53 [0.18-0.73] | | | - | | - | - |
| CCL5 | rs2280788 | CG | | C | | 6 | | | 1275 | | 1407 | | | 1.21 [0.85-1.74] | | 0.287 | 0.25 [0.00-0.68] | | | - | | - | - |
| CCL5 | rs2107538 | AG | | T | | 8 | | | 2017 | | 2107 | | | 0.81 [0.60-1.10] | | 0.182 | 0.78 [0.56-0.89] | | | - | | - | - |
| CD14 | rs2569190 | CT | | A | | 6 | | | 1072 | | 1327 | | | 0.88 [0.75-1.04] | | 0.130 | 0.00 [0.00-0.49] | | | - | | - | - |
| CD209 | rs2287886 | AG | | A | | 4 | | | 1082 | | 1243 | | | 0.99 [0.77-1.28] | | 0.968 | 0.48 [0.00-0.83] | | | - | | - | - |
| CD209 | rs735239 | AG | | G | | 4 | | | 1206 | | 1320 | | | 0.62 [0.44-0.87] | | 0.006 | 0.67 [0.03-0.89] | | | BCC | | 0.757 | 0.994 |
| CD209 | rs4804803 | AG | | G | | 8 | | | 1735 | | 1956 | | | 0.96 [0.74-1.24] | | 0.750 | 0.65 [0.25-0.84] | | | - | | - | - |
| IFNG | rs1861494 | AG | | C | | 5 | | | 2168 | | 2528 | | | 1.21 [0.98-1.49] | | 0.077 | 0.50 [0.00-0.82] | | | - | | - | - |
| IFNG | rs2430561 | AT | | A | | 23 | | | 4188 | | 4655 | | | 0.87 [0.77-0.98] | | 0.025 | 0.37 [0.00-0.62] | | | BBC | | 0.857 | 0.997 |
| IL10 | rs1800871 | CT | | A | | 13 | | | 3580 | | 5203 | | | 1.16 [0.94-1.43] | | 0.173 | 0.74 [0.55-0.85] | | | - | | - | - |
| IL10 | rs1800872 | AC | | T | | 14 | | | 3322 | | 5170 | | | 0.95 [0.80-1.12] | | 0.517 | 0.59 [0.26-0.77] | | | - | | - | - |
| IL10 | rs1800896 | AG | | C | | 23 | | | 4893 | | 6811 | | | 0.86 [0.75-1.00] | | 0.046 | 0.51 [0.21-0.70] | | | BCC | | 0.910 | 0.998 |
| IL12B | rs3212227 | AC | | G | | 8 | | | 2128 | | 2363 | | | 1.02 [0.88-1.17] | | 0.833 | 0.20 [0.00-0.62] | | | - | | - | - |
| IL18 | rs1946518 | AC | | T | | 5 | | | 1267 | | 1905 | | | 0.97 [0.84-1.12] | | 0.674 | 0.00 [0.00-0.30] | | | - | | - | - |
| IL1B | rs1143634 | [CT] | | A | | 6 | | | 954 | | 1008 | | | 0.95 [0.62-1.47] | | 0.827 | 0.65 [0.16-0.86] | | | - | | - | - |
| IL1B | rs16944 | [CT] | | A | | 6 | | | 1509 | | 1725 | | | 0.92 [0.79-1.07] | | 0.274 | 0.00 [0.00-0.69] | | | - | | - | - |
| IL2 | rs2069762 | GT | | C | | 5 | | | 566 | | 1205 | | | 1.17 [0.86-1.60] | | 0.310 | 0.49 [0.00-0.81] | | | - | | - | - |
| IL4 | rs2243250 | CT | | T | | 7 | | | 1134 | | 1816 | | | 0.96 [0.66-1.39] | | 0.820 | 0.78 [0.54-0.89] | | | - | | - | - |
| IL6 IL6R | rs1800795 | CG | | C | | 4 | | | 712 | | 944 | | | 0.65 [0.37-1.13] | | 0.126 | 0.55 [0.00-0.85] | | | - | | - | - |
| MBL2 | rs1800450 | AG (AB) | | T | | 5 | | | 622 | | 951 | | | 0.97 [0.73-1.28] | | 0.816 | 0.18 [0.00-0.83] | | | - | | - | - |
| MBL2 | rs7096206 | CG (XY) | | G | | 5 | | | 1589 | | 3079 | | | 1.05 [0.80-1.38] | | 0.719 | 0.60 [0.00-0.85] | | | - | | - | - |
| P2RX7 | rs2393799 | CT | | T | | 11 | | | 2465 | | 3047 | | | 1.03 [0.89-1.18] | | 0.726 | 0.22 [0.00-0.61] | | | - | | - | - |
| P2RX7 | rs3751143 | AC | | C | | 13 | | | 2263 | | 2961 | | | 1.30 [1.06-1.59] | | 0.011 | 0.56 [0.17-0.76] | | | BCC | | 0.758 | 0.994 |
| SLC11A1 | rs17235409 | AG | | A | | 26 | | | 3891 | | 4278 | | | 1.15 [0.95-1.39] | | 0.150 | 0.60 [0.39-0.74] | | | - | | - | - |
| SLC11A1 | rs17235416 | +/del | | - | | 23 | | | 4357 | | 4965 | | | 1.20 [1.04-1.39] | | 0.014 | 0.47 [0.13-0.67] | | | BBC | | 0.804 | 0.995 |
| SLC11A1 | rs3731865 | CG | | C | | 16 | | | 2472 | | 2787 | | | 1.24 [1.00-1.54] | | 0.051 | 0.52 [0.15-0.73] | | | - | | - | - |
| TIRAP | rs8177374 | CT | | T | | 10 | | | 6483 | | 7643 | | | 0.82 [0.62-1.10] | | 0.184 | 0.69 [0.39-0.84] | | | - | | - | - |
| TLR2 | rs5743708 | AG | | A | | 5 | | | 1631 | | 1562 | | | 1.65 [0.63-4.30] | | 0.309 | 0.57 [0.00-0.84] | | | - | | - | - |
| TLR2 | rs3804099 | CT | | C | | 5 | | | 1361 | | 1449 | | | 0.97 [0.75-1.25] | | 0.814 | 0.61 [0.00-0.85] | | | - | | - | - |
| TLR4 | rs4986791 | CT | | T | | 4 | | | 1133 | | 1104 | | | 1.08 [0.82-1.43] | | 0.591 | 0.00 [0.00-0.71] | | | - | | - | - |
| TLR4 | rs4986790 | AG | | G | | 7 | | | 1714 | | 2008 | | | 1.06 [0.86-1.31] | | 0.579 | 0.00 [0.00-0.70] | | | - | | - | - |
| TNFA | rs1800630 | AC | | A | | 4 | | | 918 | | 1124 | | | 0.86 [0.70-1.04] | | 0.124 | 0.00 [0.00-0.66] | | | - | | - | - |
| TNFA | rs1799724 | CT | | T | | 5 | | | 983 | | 840 | | | 1.35 [0.75-2.42] | | 0.313 | 0.83 [0.62-0.93] | | | - | | - | - |
| TNFA | rs361525 | AG | | A | | 12 | | | 1398 | | 2239 | | | 1.29 [0.81-2.05] | | 0.283 | 0.71 [0.49-0.84] | | | - | | - | - |
| TNFA | rs1800629 | AG | | A | | 19 | | | 2527 | | 3722 | | | 1.08 [0.90-1.31] | | 0.409 | 0.35 [0.00-0.62] | | | - | | - | - |
| VDR | rs7975232 | Aa | | A | | 8 | | | 909 | | 1394 | | | 1.04 [0.78-1.39] | | 0.793 | 0.56 [0.02-0.80] | | | - | | - | - |
| VDR | rs1544410 | Bb | | T | | 12 | | | 1576 | | 1794 | | | 1.11 [0.90-1.37] | | 0.310 | 0.40 [0.00-0.69] | | | - | | - | - |
| VDR | rs731236 | Tt | | G | | 18 | | | 2737 | | 3465 | | | 1.12 [0.95-1.33] | | 0.177 | 0.37 [0.00-0.64] | | | - | | - | - |
| VDR | rs2228570 | Ff | | A | | 19 | | | 2170 | | 2620 | | | 0.87 [0.70-1.07] | | 0.183 | 0.62 [0.38-0.77] | | | - | | - | - |
| **Influenza** |  |  | |  | |  | | |  | |  | | |  | |  |  | | |  | |  |  |
| There were no SNPs with 4 or more studies used for meta-analysis | | | | | | | | | | | | | | | | | | | | | | | |
| **Respiratory syncytial virus** | |  |  | |  | | | |  | | |  | |  | |  | |  | | |  | - | - |
| CCL5 | rs2107538 | [AG] | T | | 4 | | | | 892 | | | 1601 | | 1.06 [0.79-1.42] | | 0.706 | | 0.56 [0.00-0.85] | | | - | - | - |
| CD14 | rs2569190 | CT | A | | 5 | | | | 814 | | | 1658 | | 0.88 [0.68-1.14] | | 0.321 | | 0.38 [0.00-0.77] | | | - | - | - |
| IL4 | rs2243250 | CT | T | | 4 | | | | 896 | | | 2054 | | 1.04 [0.74-1.47] | | 0.803 | | 0.71 [0.16-0.90] | | | - | - | - |
| IL8 | rs4073 | AT | T | | 4 | | | | 1193 | | | 1937 | | 1.02 [0.81-1.27] | | 0.894 | | 0.49 [0.00-0.83] | | | - | - | - |
| TLR4 | rs4986791 | [CT] | T | | 5 | | | | 1039 | | | 3752 | | 2.97 [0.37-23.85] | | 0.306 | | 0.98 [0.97-0.99] | | | - | - | - |
| TLR4 | rs4986790 | [AG] | G | | 7 | | | | 1363 | | | 9266 | | 1.78 [0.46-6.95] | | 0.404 | | 0.96 [0.95-0.98] | | | - | - | - |
| **SARS-Coronavirus** |  |  |  | |  | | | |  | | |  | |  | |  | |  | | |  |  |  |
| There were no SNPs with 4 or more studies used for meta-analysis | | | | | | | | | | | | | | | | | | | | | | | |
| **Pneumonia** |  |  |  | | | |  |  | |  | | |  | |  | | |  |  | | |  |  |
| FCGR2A | rs1801274 | AG | G | | | | 6 | 1704 | | 2211 | | | 0.74 [0.56-0.97] | | 0.031 | | | 0.58 [0.00-0.83] | BCC | | | 0.869 | 0.997 |
| IL6 IL6R | rs1800795 | CG | C | | | | 6 | 3062 | | 2778 | | | 0.96 [0.86-1.07] | | 0.475 | | | 0.05 [0.00-0.76] | - | | | - | - |

Supplementary Table 7. Subset analysis and disease severity results, various genetic models

| Gene | rs code | Heterozygote | Risk allele | N studies | N cases (allelels) | N Controls (allelels) | OR [95% CI] | | P | I^2^ [95% CI] | Venice score | BFDP (med/low) | BFDP (very low) |
| --- | --- | --- | --- | --- | --- | --- | --- | --- | --- | --- | --- | --- | --- |
| **Disease susceptibility in controls positive on tuberculosis skin test** | | | | | | | | | | | | | |
| **Allelic model** | | | | | | | | | | | | | |
| TNFA | rs1800629 | AG | A | 5 | 1314 | 968 | 1.50 [0.98-2.30] | | 0.064 | 0.40 [0.00-0.78] | - | - | - |
| IL10 | rs1800896 | AG | C | 6 | 4780 | 4724 | 1.00 [0.89-1.13] | | 0.983 | 0.10 [0.00-0.77] | - | - | - |
| SLC11A1 | rs17235416 | +/del | - | 4 | 1366 | 706 | 0.78 [0.39-1.57] | | 0.494 | 0.70 [0.13-0.89] | - | - | - |
| SLC11A1 | rs17235409 | AG | A | 5 | 1766 | 1100 | 1.03 [0.78-1.36] | | 0.818 | 0.22 [0.00-0.68] | - | - | - |
| **Dominant model** | | | | | | | | | | | | | |
| TNFA | rs1800629 | AG | A | 4 | 517 | 403 | 1.52 [0.44-5.33] | | 0.510 | 0.00 [0.00-0.45] | - | - | - |
| IL10 | rs1800896 | AG | C | 5 | 2250 | 2281 | 1.00 [0.70-1.41] | | 0.981 | 0.60 [0.00-0.85] | - | - | - |
| SLC11A1 | rs17235416 | +/del | - | 4 | 683 | 353 | 0.99 [0.56-1.77] | | 0.986 | 0.44 [0.00-0.81] | - | - | - |
| **Recessive model** | | | | | | | | | | | | | |
| TNFA | rs1800629 | AG | A | 4 | 517 | 403 | 0.57 [0.37-0.86] | | 0.008 | 0.06 [0.00-0.86] | CAC | 0.820 | 0.996 |
| IL10 | rs1800896 | AG | C | 5 | 2250 | 2281 | 0.99 [0.80-1.24] | | 0.964 | 0.00 [0.00-0.66] | - | - | - |
| SLC11A1 | rs17235409 | AG | A | 5 | 883 | 550 | 1.38 [0.72-2.65] | | 0.327 | 0.77 [0.44-0.91] | - | - | - |
| **Heterozygote advantage** | | | | | | | | | | | | | |
| TNFA | rs1800629 | AG | A | 4 | 517 | 403 | 1.72 [1.14-2.61] | | 0.010 | 0.00 [0.00-0.79] | CAC | 0.845 | 0.997 |
| IL10 | rs1800896 | AG | C | 5 | 2250 | 2281 | 0.93 [0.65-1.35] | | 0.717 | 0.68 [0.18-0.88] | - | - | - |
| SLC11A1 | rs17235416 | +/del | - | 4 | 683 | 353 | 0.62 [0.43-0.89] | | 0.010 | 0.00 [0.00-0.71] | CAC | 0.815 | 0.996 |
| SLC11A1 | rs17235409 | AG | A | 5 | 883 | 550 | 0.65 [0.35-1.23] | | 0.187 | 0.75 [0.38-0.90] | - | - | - |
| **Disease susceptibility in exposed controls** | | | | | | | | | | | | | |
| **Allelic model** | | | | | | | | | | | | | |
| CCL2 (MCP1) | rs1024611 | AG | G | 4 | 6132 | 7112 | 0.88 [0.51-1.50] | 0.640 | | 0.97 [0.95-0.98] | - | - | - |
| IFNG | rs2430561 | AT | A | 5 | 3052 | 1938 | 1.59 [0.73-3.43] | 0.241 | | 0.97 [0.95-0.98] | - | - | - |
| **Dominant model** | | | | | | | | | | | | | |
| CCL2 (MCP1) | rs1024611 | AG | G | 4 | 3066 | 3556 | 0.85 [0.50-1.45] | 0.560 | | 0.94 [0.87-0.97] | - | - | - |
| IFNG | rs2430561 | AT | A | 4 | 1113 | 651 | 1.08 [0.89-1.32] | 0.432 | | 0.00 [0.00-0.00] | - | - | - |
| **Recessive model** | | | | | | | | | | | | | |
| CCL2 (MCP1) | rs1024611 | AG | G | 4 | 3066 | 3556 | 1.25 [0.56-2.80] | 0.584 | | 0.94 [0.87-0.97] | - | - | - |
| IFNG | rs2430561 | AT | A | 4 | 1113 | 651 | 0.67 [0.48-0.94] | 0.019 | | 0.00 [0.00-0.00] | BAC | 0.859 | 0.997 |
| **Heterozygote advantage** | | | | | | | | | | | | | |
| CCL2 (MCP1) | rs1024611 | AG | G | 4 | 3066 | 3556 | 0.79 [0.72-0.88] | 1.26E-05 | | 0.00 [0.00-0.83] | BAC | 0.014 | 0.426 |
| IFNG | rs2430561 | AT | A | 4 | 1113 | 651 | 1.06 [0.87-1.30] | 0.569 | | 0.00 [0.00-0.73] | - | - | - |
| **Severity model** |  |  |  |  |  |  |  |  | |  |  |  |  |
| **Allelic model** | | | | | | | | | | | | | |
| IL6 IL6R | rs1800795 | CG | C | 5 | 982 | 4328 | 1.23 [0.83-1.83] | 0.301 | | 0.67 [0.15-0.87] | - | - | - |
| TNFA | rs1800629 | AG | A | 5 | 936 | 2042 | 0.96 [0.76-1.22] | 0.748 | | 0.00 [0.00-0.72] | - | - | - |
| ACE | rs4340 | D/I | D | 5 | 554 | 1530 | 0.89 [0.52-1.52] | 0.671 | | 0.82 [0.59-0.92] | - | - | - |
| MYLK | rs820336 | CT | C | 4 | 118 | 2056 | 0.96 [0.60-1.53] | 0.867 | | 0.00 [0.00-0.72] | - | - | - |
| **Dominant model** | | | | | | | | | | | | | |
| IL6 IL6R | rs1800795 | CG | C | 5 | 491 | 2164 | 1.48 [0.70-3.10] | 0.301 | | 0.60 [0.00-0.85] | - | - | - |
| TNFA | rs1800629 | AG | A | 4 | 468 | 1021 | 0.70 [0.28-1.74] | 0.446 | | 0.00 [0.00-0.58] | - | - | - |
| ACE | rs4340 | D/I | D | 5 | 277 | 765 | 1.01 [0.51-1.98] | 0.983 | | 0.72 [0.28-0.89] | - | - | - |
| MYLK | rs820336 | CT | C | 4 | 59 | 1028 | 1.19 [0.35-4.00] | 0.784 | | 0.00 [0.00-0.48] | - | - | - |
| **Recessive model** | | | | | | | | | | | | | |
| IL6 IL6R | rs1800795 | CG | C | 5 | 491 | 2164 | 0.78 [0.46-1.32] | 0.358 | | 0.58 [0.00-0.84] | - | - | - |
| TNFA | rs1800629 | AG | A | 5 | 468 | 1021 | 1.02 [0.77-1.34] | 0.892 | | 0.01 [0.00-0.79] | - | - | - |
| ACE | rs4340 | D/I | D | 5 | 277 | 765 | 1.27 [0.57-2.80] | 0.557 | | 0.75 [0.38-0.90] | - | - | - |
| MYLK | rs820336 | CT | C | 4 | 59 | 1028 | 1.03 [0.59-1.80] | 0.918 | | 0.00 [0.00-0.76] | - | - | - |
| **Heterozygote advantage** | | | | | | | | | | | | | |
| IL6 IL6R | rs1800795 | CG | C | 5 | 491 | 2164 | 1.10 [0.75-1.60] | 0.631 | | 0.38 [0.00-0.77] | - | - | - |
| TNFA | rs1800629 | AG | A | 5 | 468 | 1021 | 1.06 [0.72-1.57] | 0.752 | | 0.17 [0.00-0.83] | - | - | - |
| ACE | rs4340 | D/I | D | 5 | 277 | 765 | 0.87 [0.61-1.24] | 0.439 | | 0.25 [0.00-0.70] | - | - | - |
| MYLK | rs820336 | CT | C | 4 | 59 | 1028 | 1.02 [0.59-1.79] | 0.932 | | 0.00 [0.00-0.72] | - | - | - |

| 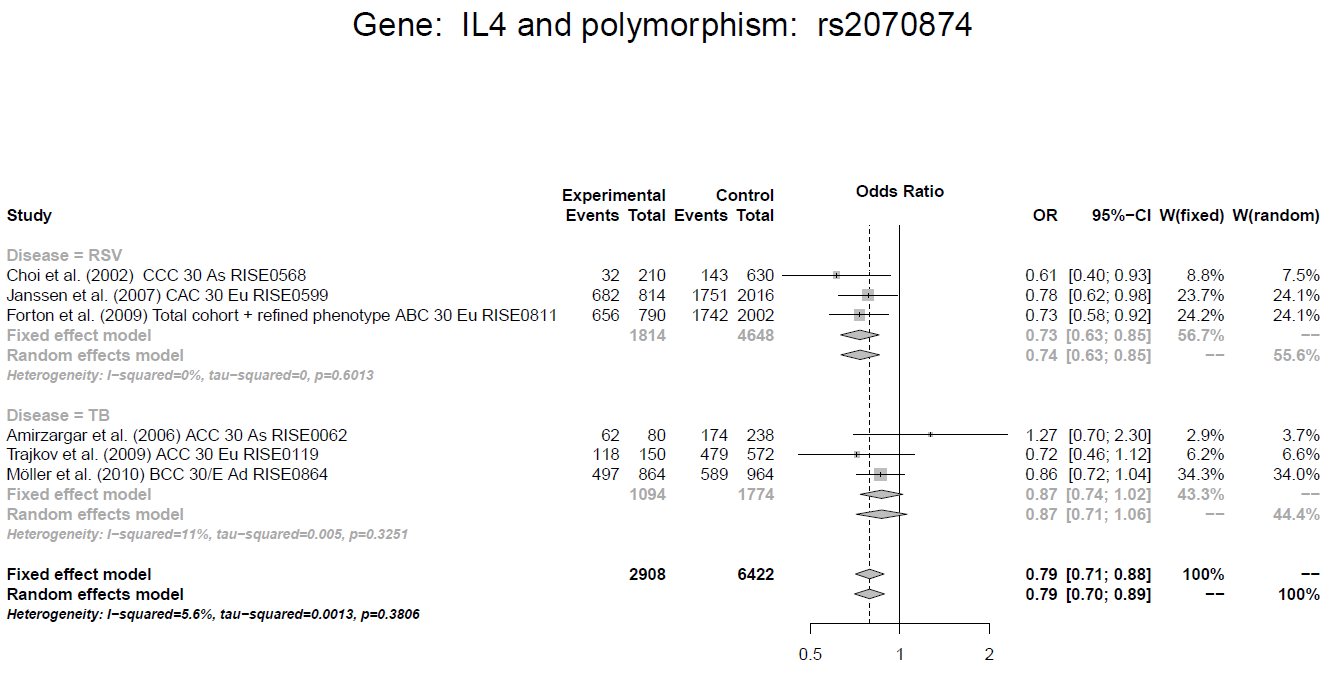  a) |
| --- |
| 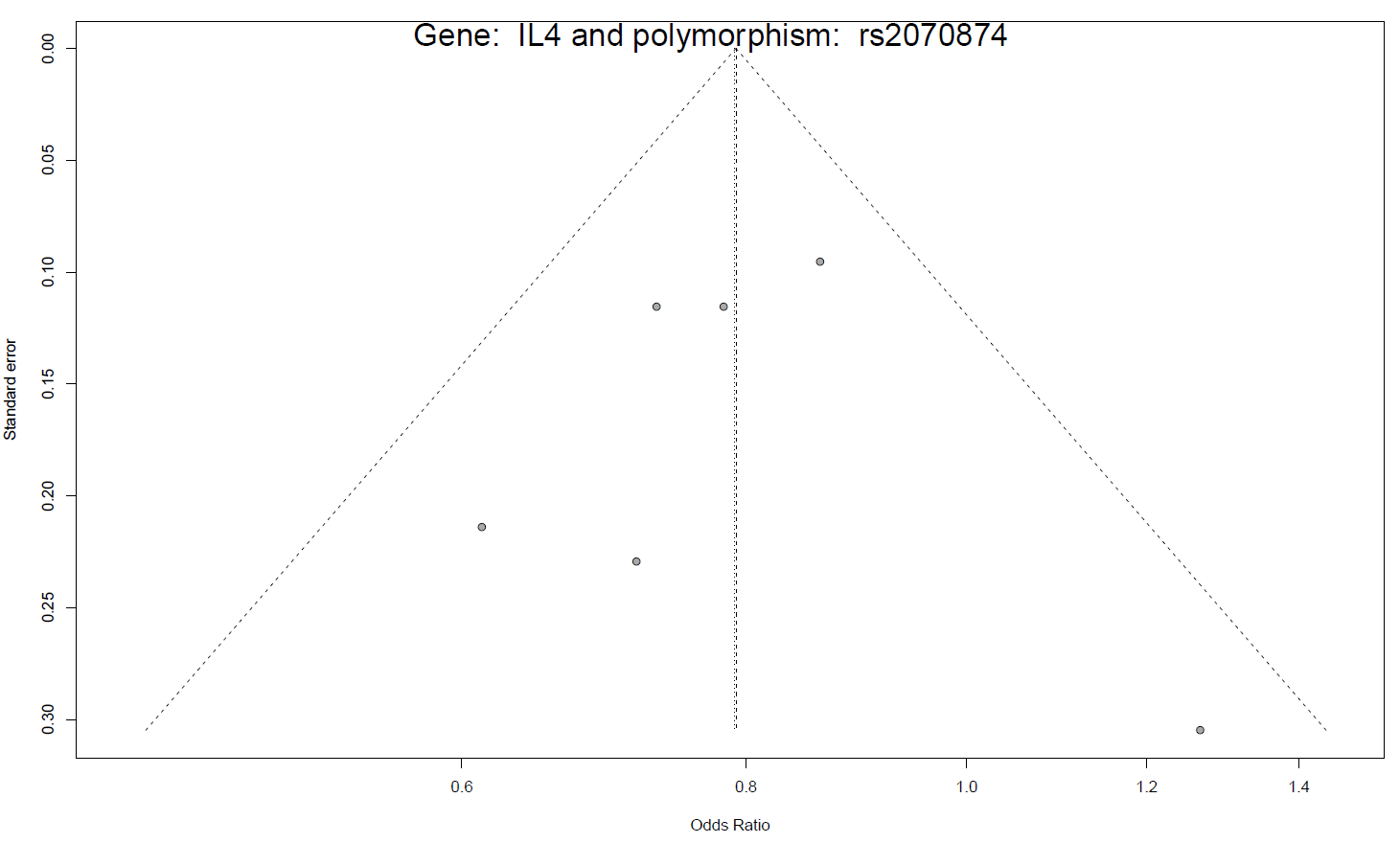  b) |

Supplementary Figure 2. Forest (a) and funnel plot (b) for allelic model for rs2070874 from IL4 gene in pooled analysis. Each line is represented by a study author and publication year, followed by the CSI score (ABC), disease model (30 denotes disease susceptibility), ethnicity (As-Asian, Af-African, Eu-European and Ad-Admixed) and lastly RISE ID, available in the RISEdb website

| 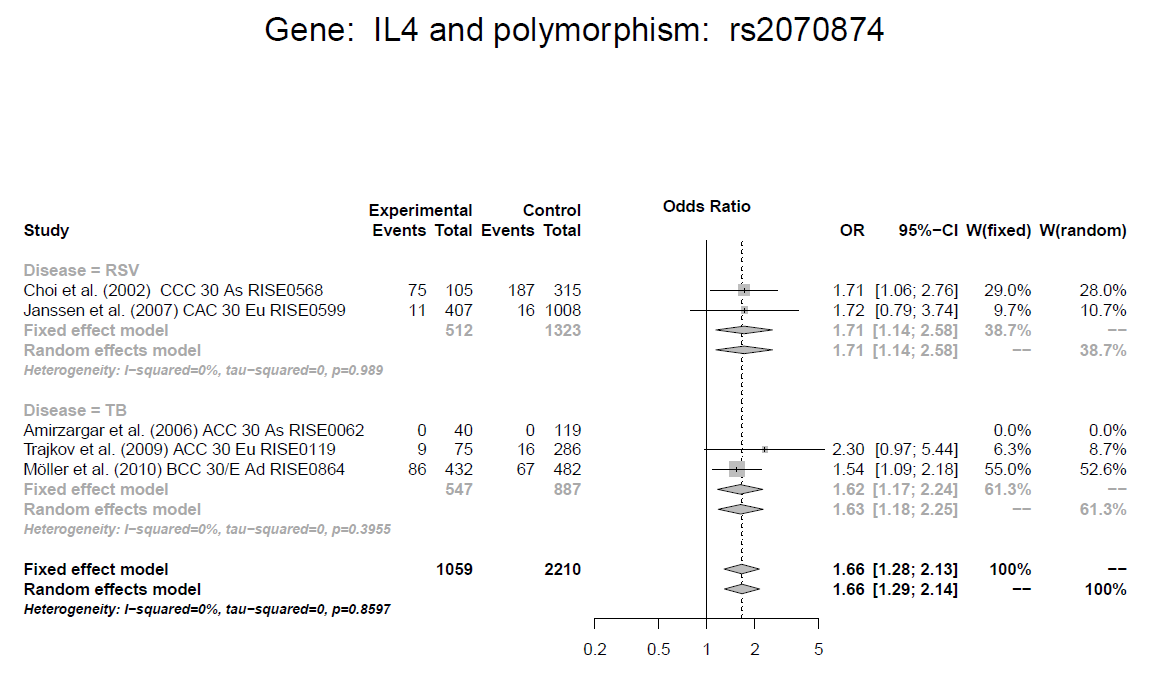  a) |
| --- |
| 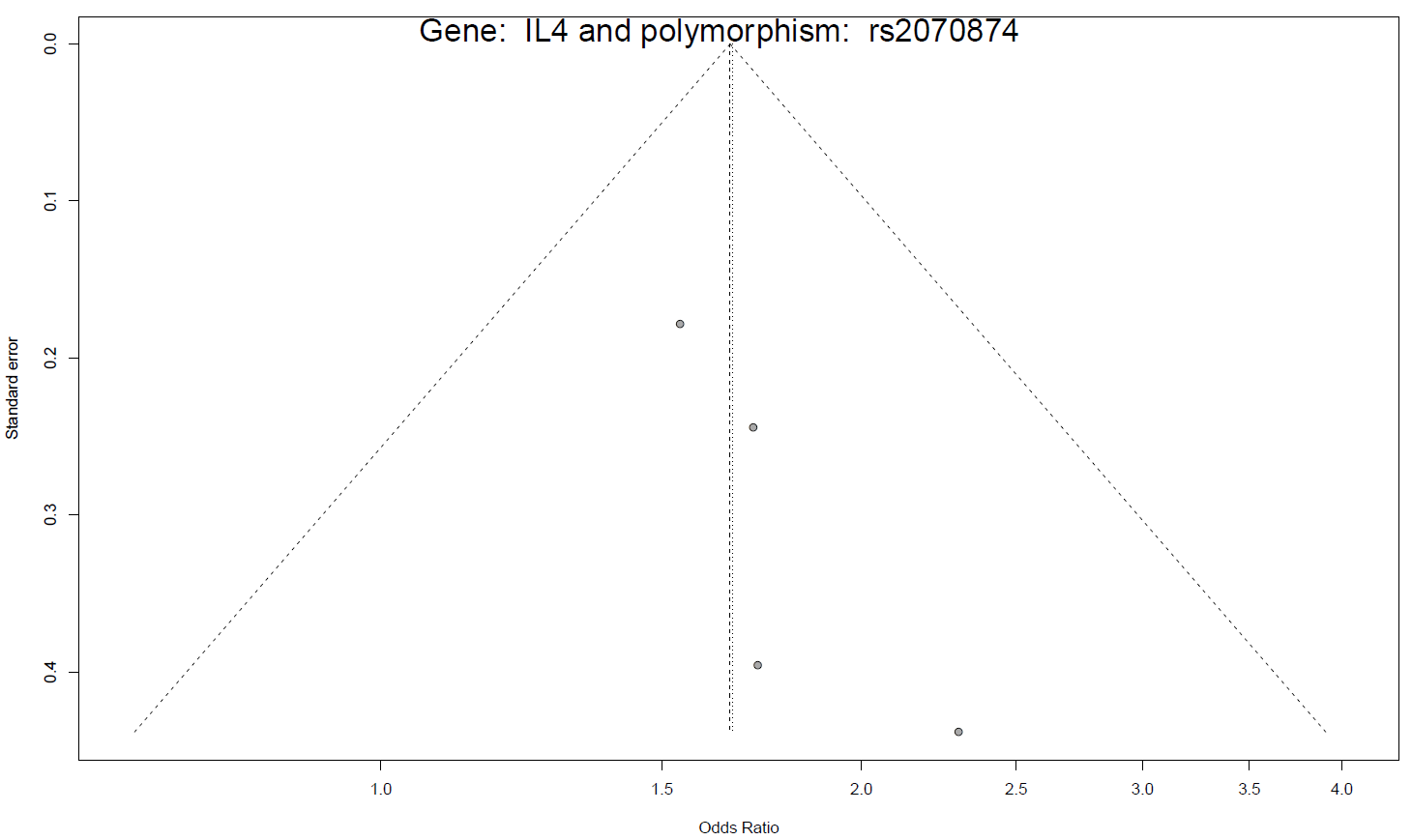  b) |

Supplementary Figure 3. Forest (a) and funnel plot (b) for recessive model for rs2070874 from IL4 gene in pooled analysis. Each line is represented by a study author and publication year, followed by the CSI score (ABC), disease model (30 denotes disease susceptibility), ethnicity (As-Asian, Af-African, Eu-European and Ad-Admixed) and lastly RISE ID, available in the RISEdb website

| 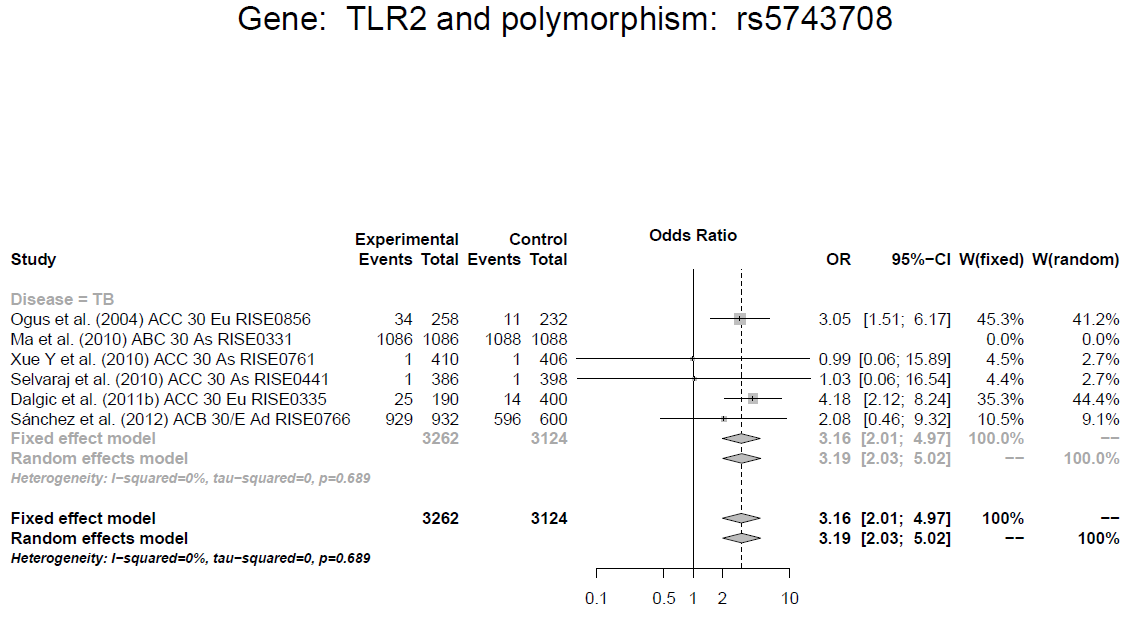  a) |
| --- |
| 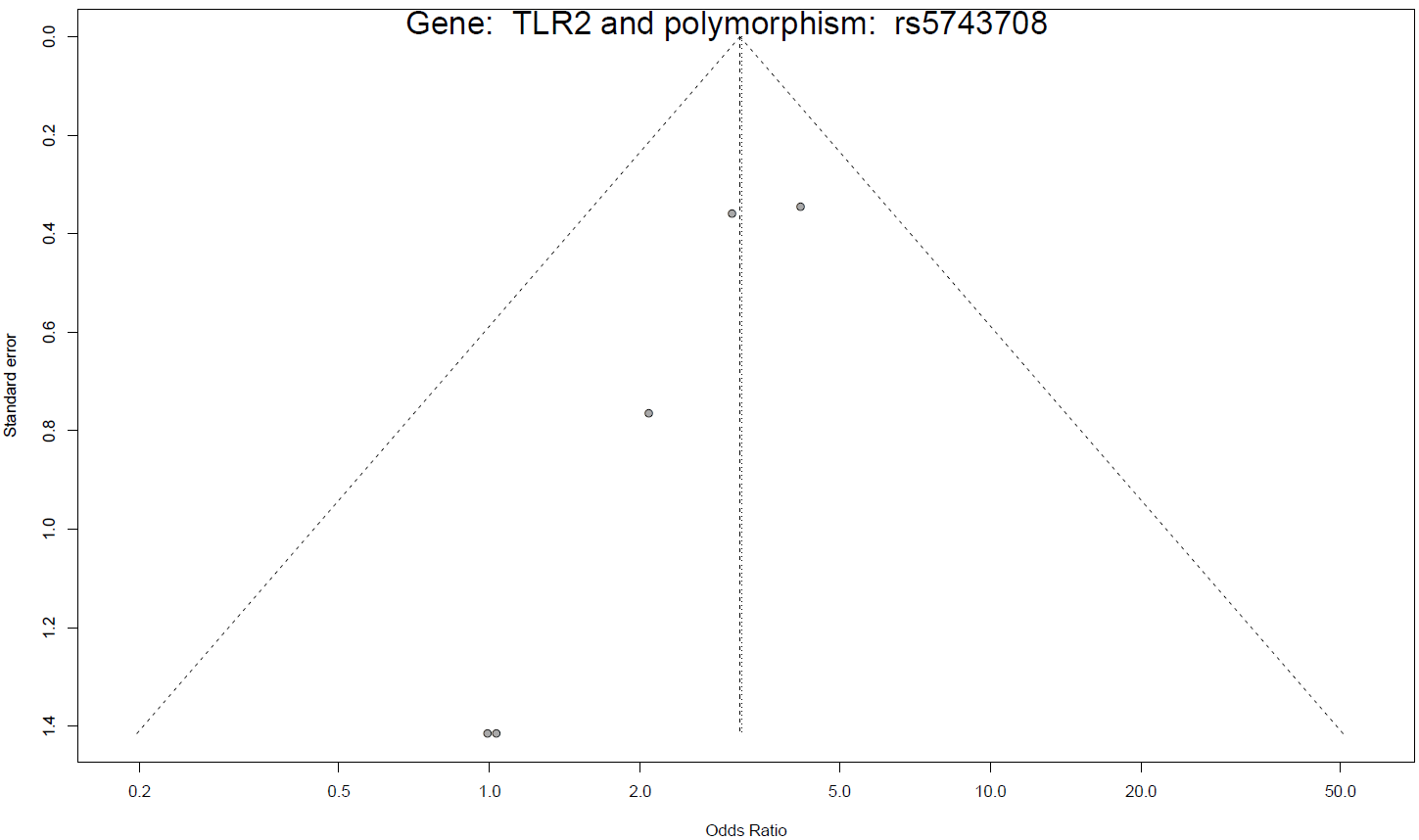  b) |

Supplementary Figure 4. Forest (a) and funnel plot (b) for allelic model for rs5743708 from TLR2 gene in tuberculosis analysis. Each line is represented by a study author and publication year, followed by the CSI score (ABC), disease model (30 denotes disease susceptibility), ethnicity (As-Asian, Af-African, Eu-European and Ad-Admixed) and lastly RISE ID, available in the RISEdb website

Supplementary Table 8. Overview of family-based and linkage studies that investigated host genetic factors for respiratory infectious diseases

| Study, pathogen; RISEdb ID | Design | Main result |
| --- | --- | --- |
| Bellamy R, et al (2000), tuberculosis; RISE0550 | Two-stage genome-wide linkage study, using sibpair families with two full siblings with tuberculosis. 299 markers were used in 92 sibpairs from The Gambia and South Africa. 22 markers from regions with likely positive hits were genotyped in a second set of 81 sibpairs from the same countries | Markers on chromosomes 15q and Xq showed suggestive evidence of linkage (LOD=2.00 and 1.77, respectively), replicated in common ancestry using microsatellite mapping |
| Cervino ACL, et al (2000), tuberculosis; RISE0474 | Family based association study (extended TDT) of NRAMP1 in families from Guinea-Conakry | Single base change in intron 4 was nominally significant (P=0.036) |
| Greenwood MT, et al (2000), tuberculosis; RISE0561 | Association analysis of region of NRAMP1 gene (chromosome 2q35) and tuberculosis in extended aboriginal Canadian family | Maximum LOD score of 3.81 is reported for marker D2S424 located just distal to NRAMP1 |
| Hull J, et al (2000), bronchiolitis; RISE0807 | 117 families tested for association between the 5' flanking region of IL-8 polymorphism and bronchiolitis | Single nucleotide polymorphism located 251bp upstream of the IL-8 transcriptional start site was detected. The frequency of this allele was significantly increased in infants with bronchiolitis (P=0.014) and particularly in those without known risk factors (P=0.004) |
| Hull J, et al (2001), bronchiolitis; RISE0564 | 77 family trios were included in TDT association analysis of IL8 haplotypes with bronchiolitis | Previously published association between IL85251A allele and bronchiolitis was confirmed |
| El Baghdai J, et al (2003), tuberculosis; RISE0033 | Family based association of 116 families from Morocco, investigating seven polymorphisms in NRAMP1 | No significant association was found between tuberculosis and any of the NRAMP1 markers |
| Hull J, et al (2003), RSV bronchiolitis; RISE0575 | Genotyping of CC chemokine receptor 5 was done in 580 cases and both parents | Two polymorphisms, -2459G and -2554T variants were associated with severe RSV bronchiolitis (P=0.01) |
| Daly KA, et al (2004), otitis media; RISE0587 | Subjects who had tympanostomy and their families were genotyped, with 121 that had at least two individuals who had received the diagnosis of COME/ROM (364 affected and genotyped individuals), of whom 238 affected and informative relative pairs were used for analyses | Chromosome 10q at marker D10S212 (LOD 3.78; P=3.0 x 10E-5) and chromosome 19q at marker D19S254 (LOD 2.61; P=5.3 x 10E-4) were detected. Additional LOD interaction item on chromosome 3p was detected (between markers D3S4545 and D3S1259), suggesting interactions between genes in several candidate regions of the genome, probably modulated by environmental risk factors |
| Jamieson SE, et al (2004), tuberculosis; RISE0518 | 92 multicase tuberculosis (627 individuals) and 72 multicase leprosy (372 individuals) families from Brazil were genotyped with 16 microsatellites. In addition, 49 informative single nucleotide polymorphisms were typed in candidate genes | Single peak for tuberculosis was detected at D17S250 (P=0.02). Combined analysis with leprosy confirmed the result (peak 3.38) at D17S250, equivalent to an allele sharing LOD score 2.48 (P=0.0004). Four loci were implied (NOS2A, CCL18, CCL4 and STAT5B), supporting hypothesis of a cluster of susceptibility genes across 17q11.2 |
| Miller EN, et al (2004), tuberculosis; RISE0519 | 405 markers were typed in 16 families (178 individuals) with tuberculosis. In second stage 58 markers from positive regions were typed in a second set of 22 (176 individuals) tuberculosis families, with 22 additional markers typed in all families | Three regions (10q26.13, 11q12.3, 20p12.1) retained suggestive evidence (peak LOD scores 1.31, 1.85, 1.78; P=0.007, 0.0018, 0.0021) for linkage to tuberculosis |
| Remus N, et al (2004), tuberculosis; RISE0583 | Transmission disequilibrium test (TDT) was used to study the association of polymorphisms within IL12RB1 gene and tuberculosis in 101 Moroccan families | Sequence analysis of all coding IL12RB1exons and their flanking intron regions, and a promoter segment revealed nineteen point mutations (9 known and 10 unknown). None of these point mutations corresponded to previously reported IL12RB1 null mutations. Two promoter polymorphisms (in strong linkage disequilibrium) were found to be associated with pulmonary tuberculosis |
| Casselbrant ML, et al (2009), otitis media; RISE0881 | Genome-wide linkage scan using the 8,802 SNPs from 10K Affymetrix panel in 403 Caucasian families containing 1,431 genotyped individuals and 377 genotyped affected sib pairs, and 26 African American families containing 75 genotyped individuals and 27 genotyped affected sib pairs | Linkage peak on chromosome 17q12 at rs226088 had a p-value of 0.00007. Other peaks of interest were 10q22.3 (0.00181 at rs1878001), 7q33 (0.00105 at rs958408), 6p25.1 (0.00261 at rs554653), and 4p15.2 (0.00301 at rs2133507). Family-based association testing revealed signals near previously implicated genes: 513 kb from SFTPA2 (10q22.3), 48 kb from IFNG (12q14), and 870 kb from TNF (6p21.3). However, no support was detected for previously implied 10q26.3 and 19q13.43. Plausible candidates include AP2B1, CCL5, and a cluster of other CCL genes, and in 10q22.3, SFTPA2 |
| Mahasirimongkol S, et al (2009), tuberculosis; RISE0485 | Nonparametric linkage analysis was performed using SNP GeneChip array with 59,860 markers, in 93 Thai families with multiple siblings, 195 individuals affected with tuberculosis | Region on chromosome 5q had suggestive evidence (LOD score=2.29, P=0.0005), and two candidate regions on chromosomes 17p and 20p |
| Thomas NJ, et al (2009), RSV; RISE0907 | Transmission disequilibrium test (TDT) analysis was used to determine transmission of variants and haplotypes within surfactant protein (SP) -A and SP-D from parents to affected offspring. Study included 148 children with active RSV and one or both parents | One SP-A and one SP-D variant showed nominally significant associations with the development of severe RSV disease |
| Horby P, et al (2010), influenza; RISE0476 | 52 familial clusters of A/H5N1were examined for plausibility of a host genetic effect on susceptibility to A/H5N1 infection | 102 confirmed cases in 52 clusters were examined, out of which only 6 cases were non-genetically related to other cases in the cluster. Some evidence from animal models and epidemiological data on A/H5N1 transmission suggest the possible role of genetic susceptibility to A/H5N1 |
| Ridruechai C, et al (2010), tuberculosis; RISE0489 | Family based association analysis of SNPs located within chromosome 5q31 region. The study included 205 Thai family trios | 75 SNPs were analyzed in total, after passing quality control. Nominal association is reported for markers lying within SLC22A4, SLC22A5 and RAD50 genes. Haplotype analysis revealed one significant haplotype block comprising SNPs rs274559, rs274554 and rs274553 of SLC22A5 gene |
| Chen WM, et al (2011), otitis media; RISE0140 | 607 individuals from 139 families, including 159 affected sib pairs and 62 second-degree affected relative pairs, were genotyped at 1,091 SNPs. For purpose of nonparametric linkage analysis, modeling marker-to-marker linkage disequilibrium | The maximum LOD score was increased to 3.75 (P=1.6*10-5) at position 63.4 Mb. The support interval contains over 90 known genes, including several genes involved in the inflammasome protein complex, a key regulator of the innate immune response to harmful exogenous or endogenous stimuli. The study has refined the 19q region of linkage, and association results suggest that the linkage signal may be due to rare variants |
| Rye MS (2011), otitis media; RISE0491 | Family based association analysis between two genes (FBXO11, EVI1) and recurrent acute otitis media (rAOM) and chromic OM with effusion (COME) susceptibility | Discovery and subsequent replication analysis revealed significant association with two polymorphism in FBXO11 gene (meta-analysis- rs12712997, P=1.41x10^-5^; rs330787, P=2.98x10^-6^). No significant associations were found for markers within EVI1 gene |
| Rye MS (2013), otitis media; RISE0403 | SLC11A1 polymorphisms were tested for association with otitis media in early childhood. 660 affected individuals from 531 families were included | Four polymorphic variants in the human SLC11A1 gene showed nominal significance with susceptibility to OM in predominantly Caucasian cohort. Haplotype analyses support a single genetic effect in the proximal region of SLC11A1 |

Supplementary Table 9. Overview of genome-wide association studies that investigated host genetic factors for respiratory infectious diseases

| Study, pathogen, ID | Design | Main result |
| --- | --- | --- |
| Davila S, et al (2008), tuberculosis; RISE0913 | 439 cases and 490 controls from Indonesia; 149 SNPs from 18 candidate genes; replication performed in Russian cohort (cases=1,873, controls=1,779) | Four SNPs in the TLR8 gene on chr X showed evidence of association with TB susceptibility in males, including a non-synonymous polymorphism rs3764880 (P=0.007, OR=1.8). Replication in an independent Russian dataset showed the SNP was nominally significant in males (P=0.03, OR=1.2) |
| Thye T, et al (2010), tuberculosis; RISE0504 | Gambian and Ghanaian case-control datasets; 333,754 SNPs were included in the meta-analysis of 2,237 cases and 3,112 controls. Additional 1,076 cases and 1,611 controls form Ghana were used to type top 17 SNPs from the meta-analysis. Further two most significant SNPs from the joint meta-analysis of discovery and replication cohorts were typed in smaller Ghanaian (150 cases, 779 controls) and Malawi cohorts (case=236, controls=779) | Combined meta-analysis of the discovery and first Ghanaian replication cohort yielded two SNPs with p<5x10^-7^(rs2335704, rs4331426). Combined meta-analysis of all stages yielded one genome wide significant result (rs4331426, P=6.8x10^-9^, OR =1.19). Marker is located on chromosome 18 in a gene desert region |
| Mahasirimongkol S, et al (2012), tuberculosis; RISE0497 | 433 Thai patients and 295 healthy controls, replicated in 188 Japanese cases and 934 controls. These two populations were meta-analyzed on 533,252 overlapping SNPs between the two studies. This was followed by a replication of the top 25 SNPs from the meta-analysis in replication samples of Thai (369 cases, 49 controls) and Japanese (112 cases, 1,089 controls) datasets | No genome wide significant result. Stratification by the age of onset meta-analysis yielded one genome wide significant locus in the young (<45 years) dataset (rs6071980, P=2.51x10^-8^, OR=1.73) on chromosome 20 between MAFB and HSEPEP1 genes |
| Png E, et al (2012), tuberculosis; RISE0885 | Three staged GWAS in Indonesian population; first stage included a genome scan of ~100,000 markers in 125 cases and 134 controls. Second stage involved genotyping of 2,453 highest ranking SNPs from stage 1 in 626 cases and 563 controls. Stage three involved replication of 251 most significant SNPs from first two stages in independent Russian cohort of 1,837 cases and 1,779 controls | Suggestive evidence of association between 8 markers and tuberculosis susceptibility was presented. All eight loci showed nominal significance in all three stages of the analysis with consistent effects in all three tested populations. Most significant marker in the overall meta-analysis was rs2273061 (P=0.0004, OR=1.16) on chromosome 20 located in the transcript of JAG1 |
| Rye MS, et al (2012), otitis media; RISE0953 | 416 cases and 1,075 controls used in the genome wide association analysis of imputed genotypes form West Australian Pregnancy Cohort Study | No genome wide significant result. None of the markers achieved required significance threshold (P< 0.002) |
| Thye T, et al (2012), tuberculosis (see Thye et al 2010); RISE0502 | Imputation of data from the 1000 Genomes project to Ghanian individuals. Top results (p<10-5) were genotyped in the same dataset. Only 11 markers showed p values <10-5 in the genotyped dataset. These SNPs were genotyped in the replication sample of 817 cases and 3,805 controls form Ghana. Top ranking SNP was genotyped in additional cohort of 1,207 cases and 1,349 individuals form Gambia, 1,025 cases and 983 controls form Indonesia, and 4,441 cases and 5,874 controls from Russia | One genome-wide significant result was detected in the combined meta-analysis of the discovery and first stage replication samples (rs2057178, P=2.63x10^-9^, OR=0.77). Further replication in three additional cohorts showed consistent effect of the allele, and nominal significance in two out of tree population, with combined meta-analysis p-value from all five datasets reaching 2.57x10^-11^. This SNP is located in an intergenic region downstream of WT1 gene |
| Allen KE, et al (2013), otitis media; RISE0227 | 602 subjects form 143 families, replicated in 1,584 individuals from 441 families | No genome wide significant result. Top ranked marker rs1110060 (P=9.1x10^-7^, OR=0.51) is located on chr 15 within the KIF7 gene. Top 45 SNPs were genotyped in a replication cohort, and one SNP on chromosome 2 reached significance threshold (rs10487394, p=2.9x10^-5^) |
| Chimusa ER, et al (2013), tuberculosis; RISE0634 | 642 cases and 91 controls of South African Coloureds. Data were genotyped on 500K Affymetrix chip and imputed using both HapMap3 and 1000 Genomes project populations. Replication of the previously reported genome wide significant SNPs (from Thye et al. 2010, 2012; Davilla et al. 2008) | No genome wide significant result. Study did confirm previously reported signals on chr 11 for WT1 gene (rs2057178, P=2.71x10^-06^, OR=0.62) but failed to replicate the signal on chromosome 18q11.2 and results from Davilla et al. 2008 on chr X |
| Rye MS, et al (2014), otitis media; RISE0887 | 256 cases and 575 controls were used for fine mapping of the 10q26.3 region using 10,185 genotyped and imputed SNPs across the region | Reported top SNPs within genes were rs7902734 (P=8.04x10^-4^; ADAM12), rs9418832 (P=7.48x10^-5^; DOCK1) and rs7922424 (P=9.47x10^-6^; intergenic between TCERGIL and PPP2R2D) |
| Chen Y, et al. (2015), influenza; RISE2022 | 102 A(H7N9) patients and 106 heavily-exposed healthy poultry workers | The rs4820294/rs2899292 haplotype GG, in association with protection from A(H7N9) infection (OR = 0.26, P = 5.92 × 10(-7)). Additionally, rs4820294 was mapped as an eQTL in human primary monocytes and lung tissues |
| Curtis J, et al. (2015), tuberculosis; RISE2029 | 5,530 people with pulmonary TB and in 5,607 healthy controls | ASAP1 gene on chromosome 8q24 (P = 2.6 × 10(-11) for rs4733781; P = 1.0 × 10(-10) for rs10956514) was associated with tuberculosis |

Supplementary Table 10. Cryptic controls

One of the biggest problems of previously published studies arises as the consequence of controls definition. It is this stage that defines the underlying *cryptic* hypothesis of the study, as control diagnostics process often can’t provide an answer to the proposed hypothesis. Firstly, we must deal with controls exposed to the same diagnostic protocol as cases do (the use of “healthy” control should be completely abandoned). Even then can we claim to be dealing with appropriate controls, as they can suffer from a number of other possible problems. These include: misdiagnosed controls (due to errors in diagnostic process), pre-disease sampling (either sampling of controls prior to the epidemic outbreak or use of cord blood samples as controls for adult cases; in both situations controls will have nearly the same chances of becoming cases under the assumption of equal risk across the population), susceptible and unexposed controls (who would develop an infection if exposed to pathogen), insufficiently exposed and susceptible (for less virulent pathogens, where infectivity in dose-effect is present), susceptible and vaccinated (and thus effectively resistant), those with undetected latent infections (when controls assignment process is partial or superficial or we are lacking proper tools to diagnose them), and finally mild disease cases (who will either not recognize the disease themselves or will not require health care support). Unfortunately, any of these categories has a potential to affect effect-to-bias ratio at the several orders of magnitude, if we assume polygenic nature and very low variances explained by a single SNP. Therefore, a careful selection of controls is one of the best strategies to propel the field development.
